# Lineage Tracking the Generation of T Regulatory Cells From Microbial Activated T Effector Cells in Naïve Mice

**DOI:** 10.3389/fimmu.2019.03109

**Published:** 2020-01-17

**Authors:** Kun Zhu, Chenfeng He, Si-Qi Liu, Mingjuan Qu, Tao Xie, Xiaofeng Yang, Lei Lei, Xiaobo Zhou, Lin Shi, Dan Zhang, Yanbin Cheng, Yae Sun, Huiqiang Zheng, Xiaonan Shen, Qijing Li, Ning Jiang, Baojun Zhang

**Affiliations:** ^1^Department of Pathogenic Microbiology and Immunology, School of Basic Medical Sciences, Xi'an Jiaotong University, Xi'an, China; ^2^Department of Biomedical Engineering, The University of Texas at Austin, Austin, TX, United States; ^3^Department of Immunology, Duke University Medical Center, Durham, NC, United States; ^4^College of Life Sciences, Ludong University, Yantai, China; ^5^Institute for Cellular and Molecular Biology, The University of Texas at Austin, Austin, TX, United States

**Keywords:** tolerance, inducible Tregs, microbiota, gut, TCR repertoire

## Abstract

Regulatory T cells (Tregs) are essential for the maintenance of gut homeostasis by suppressing conventional CD4^+^ helper T cells (Tconvs) that are activated by microbial antigens. Although thymus is the major source of the peripheral Tregs, peripheral conversion from Tconvs to Tregs have also been shown to occur under various experimental conditions. It remains less clear about the frequency of lineage conversion from Tconvs to Tregs in naïve animals. Here we used a newly established reporter system to track a group of post expansion Tregs (eTregs), which exhibited a stronger suppressive ability than the non-lineage marked Tregs. Notably, microbial antigens are the primary driver for the formation of eTregs. TCR repertoire analysis of Peyer's patch T cells revealed that eTregs are clonally related to Tconvs, but not to the non-lineage tracked Tregs. Adoptive transfer of Tconvs into lymphopenic hosts demonstrated a conversion from Tconvs to eTregs. Thus, our lineage tracking method was able to capture the lineage conversion from microbial activated effector T cells to Tregs in naïve animals. This study suggests that a fraction of clonally activated T cells from the natural T cell repertoire exhibits lineage conversion to Tregs in response to commensal microbes under homeostatic conditions.

## Introduction

CD4^+^CD25^+^ regulatory T cells (Tregs) play indispensable roles in peripheral tolerance through suppressing excessive auto-reactive and deleterious immune responses (1, 2). They can either be generated directly from developing T cells in the thymus (nTregs) or be induced (iTregs) from conventional CD4^+^CD25^−^ T cells (Tconvs), and both nTregs and iTregs can inhibit effector T cell response (3–5).

Among many foreign antigens, microbiota imposes a major challenge to the immune system. The immune system must learn to tolerate the commensals, which naturally live with the host throughout life, while remain on alert to provide defense against incidental infections. Most T cells in the gut associated lymphoid organs such as Peyer's patch (PP) are antigen experienced even in naïve animals, suggesting that our immune system constantly sees and responds to commensals. In fact, genetic ablation of Tregs in adult animals invariably leads to inflammatory bowel disease (IBD) among many other autoimmune diseases (6–8). The importance of Tregs in maintaining immune tolerance to microbiota has also been demonstrated in the adoptive transfer model: naïve T cells upon transferring into lymphopenic hosts will undergo commensal-dependent clonal expansion and quickly cause IBD in the host (9). The IBD in this experimental model can be effectively suppressed if Tregs, either nTregs or iTregs, were transferred together with naïve T cells (10–13). Thus, immune tolerance to commensals is primarily dependent on a generic immune suppressive function provided by the Tregs.

While the involvement of Tregs in maintaining immune tolerance to commensals has been firmly established, the origin of gut resident Tregs in naïve animals remains controversial. The Tregs in the gut could be expanded from pre-existing self-reactive nTreg population and/or induced from Tconv population recognizing bacterial antigens. High-throughput sequencing analysis of TCR repertoires showed that colonic Tregs exhibited greater similarity with thymic Tregs than with Tconvs (14, 15), supporting the idea that colonic Tregs are derived from pre-existing thymic nTregs. In contrast, TCR sequencing data from a separate study showed that colonic Tregs used different TCRs compared to Tregs in other locations and these TCRs can respond to commensal antigens and do not support thymic Treg development (16). Indeed, many studies have shown that Tregs can be experimentally induced in the gut with exposure to antigens and certain environmental factors (3, 17–22). Currently, methods available for tracking lineage conversion from Tconvs to Tregs inside naïve animals under homeostatic conditions are still limited to certain effector lineages (23, 24). Therefore, to what extent microbial driven iTregs contribute to the total population of Tregs in the gut lymphoid organs still remains to be further clarified.

In this study, we employed a recently established genetic tool for tracking the descendent of activated T cells that have undergone clonal expansion. Assuming that iTreg is a result of antigen induced clonal activation of pre-existing or recently activated T effector cells, we predict that clonal activation and expansion of the T effectors should produce sister clones of post expansion iTregs and T effectors with identical TCR sequences. By examining TCR repertories of clonally expanded populations we have identified clonal descendants present in both post expansion Tregs (short as eTreg for expanded Treg) and T effector cells in the Peyer's patch of naïve animals. We provide further evidence to support the idea that microbiota play an important role in driving the development of these post expansion eTregs in the gut.

## Results

### Tlox/Ox40Cre System Tracks a Population of Post Expansion Tregs

**Figure 1:**
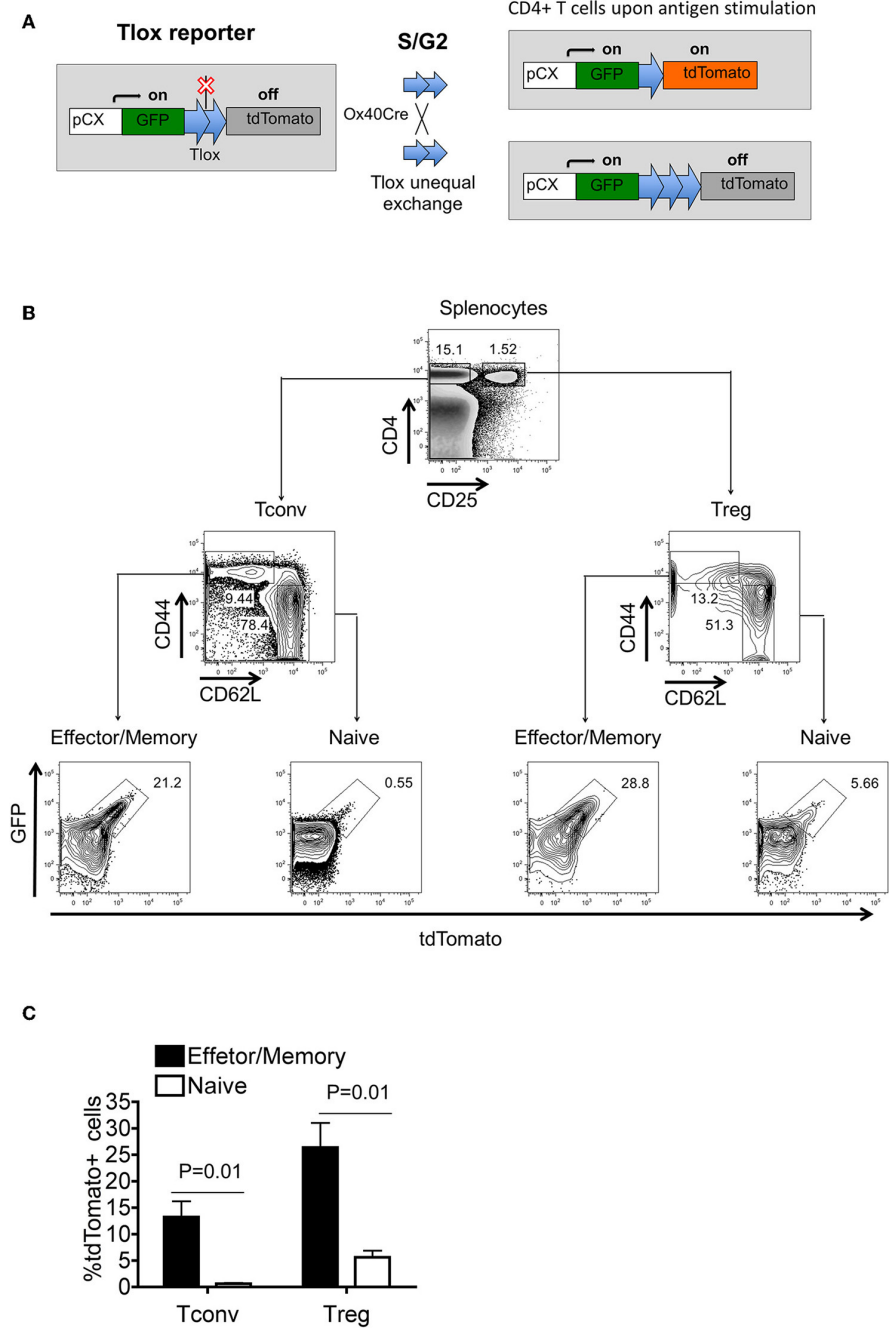
Tlox/Ox40Cre genetic system tracks regulatory T cells with proliferation history in unchallenged mice. Tlox mice were crossed with Ox40Cre transgenic mice, and tdTomato expression was analyzed in CD4^+^ cells from thymus and periphery. **(A)** Schematic diagram of Tlox/Ox40Cre combined system for tracking divided T cells. With Ox40Cre activation, tdTomato marker could only be activated in proliferating CD4^+^ T cells. **(B)** Representative FACS analysis of tdTomato expression in conventional and regulatory CD4^+^ T cells. **(C)** Statistics for **(B)** (*n* = 3). Each FACS plot represents for three independent mice.

Our previous studies have demonstrated that the Tlox system could be used to track lymphocyte proliferation in combination with appropriate Cre transgenes (25). Cre-mediated recombination between a paired Tlox sites on sister chromatids occurs exclusively during cell cycle, resulting in permanent activation of the tdTomato marker from the Tlox reporter among a fraction of the daughter cells (Figure 1A). In this study, we combined the Tlox reporter with the Ox40Cre transgene to label and track T cells when Ox40Cre is activated during T cell activation and clonal expansion. Using the R26^tdTomato^ reporter, we confirmed the published observation (26) that Ox40Cre activity is primarily restricted to peripheral Tregs and activated CD4 Tconvs (Supplemental Figures 1A,B). To evaluate the effect of Ox40Cre on activation of the Tlox reporter, we separated splenic T cells into naïve and effector fractions before examining tdTomato expression. The frequency of tdTomato activation is much higher among activated CD44^hi^ Tconvs and Tregs than their CD44^lo^ naïve counterparts (Figures 1B,C). Analysis using a reverse gating strategy further confirmed that most tdTomato labeled cells are CD44^hi^ T cells (Supplemental Figures 2A,B). The labeling frequency among T cells collected from various lymphoid organs varies from lowest in the thymus to highest in the Peyer's patch (Supplemental Figures 2C,D). The observed pattern of Tlox expression is consistent with our experimental design that Tlox activation is dependent on both T cell activation and proliferative expansion. Because tdTomato labeled cells are derived from proliferative expansion, we used the name eTregs and eTconvs (“e” as expansion) for the lineage tracked Tregs and Tconvs, respectively, in this paper.

### eTregs Exhibited Elevated Expression of Effector Genes and Enhanced Suppressor Functions

**Figure 2:**
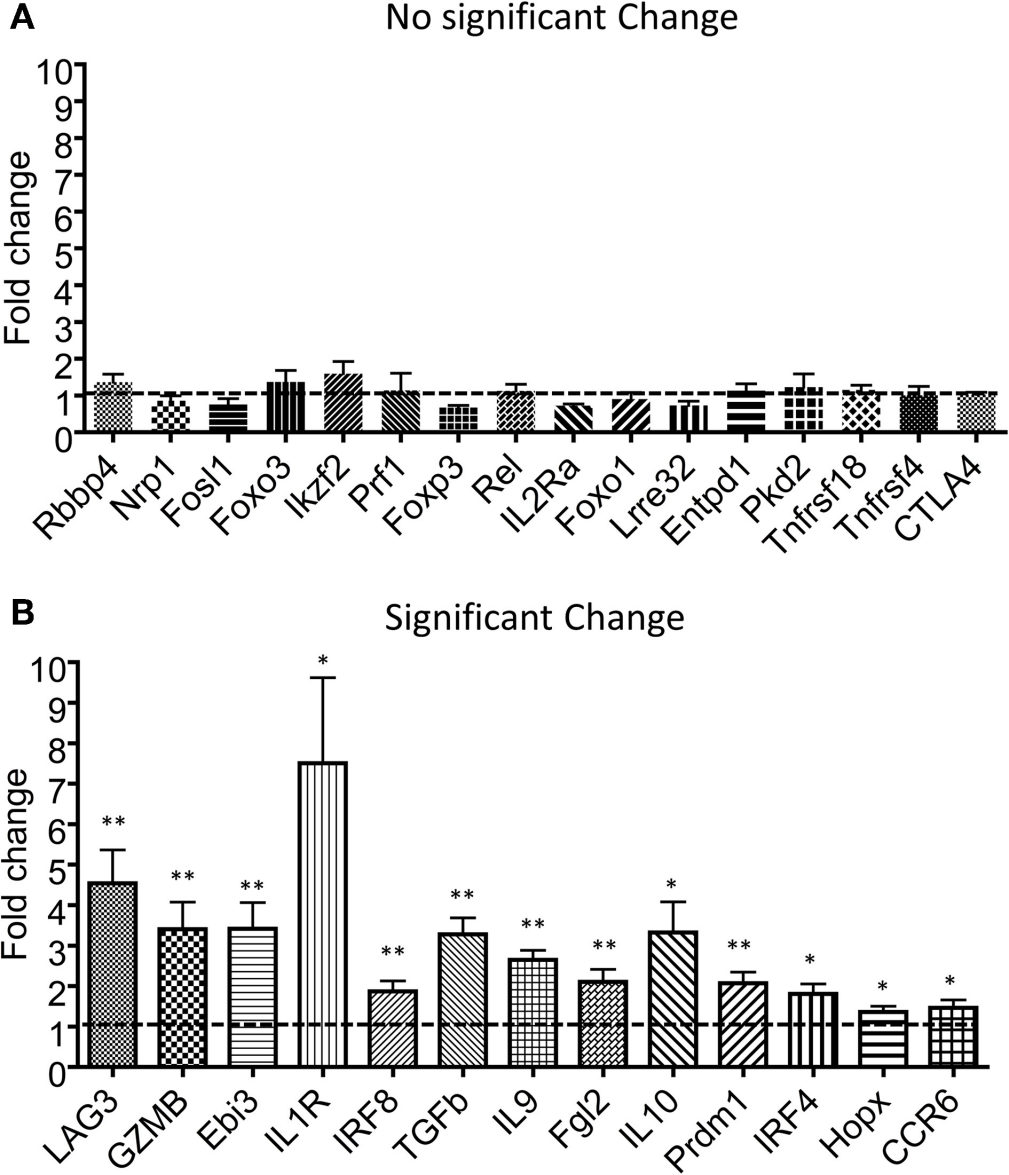
Transcription analysis for effector molecules in eTregs. The tdTomato^−^ and tdTomato^+^ eTregs were sorted from spleen of Tlox/Ox40Cre mice. After RNA extraction and cDNA synthesis, the expression of Treg-related genes was analyzed using qPCR. **(A)** The genes with no significant changes at transcriptional level. **(B)** The genes with significant changes at transcriptional level. The data includes three independent experiments in each group. *P < 0.05 and **P < 0.01.

**Figure 3:**
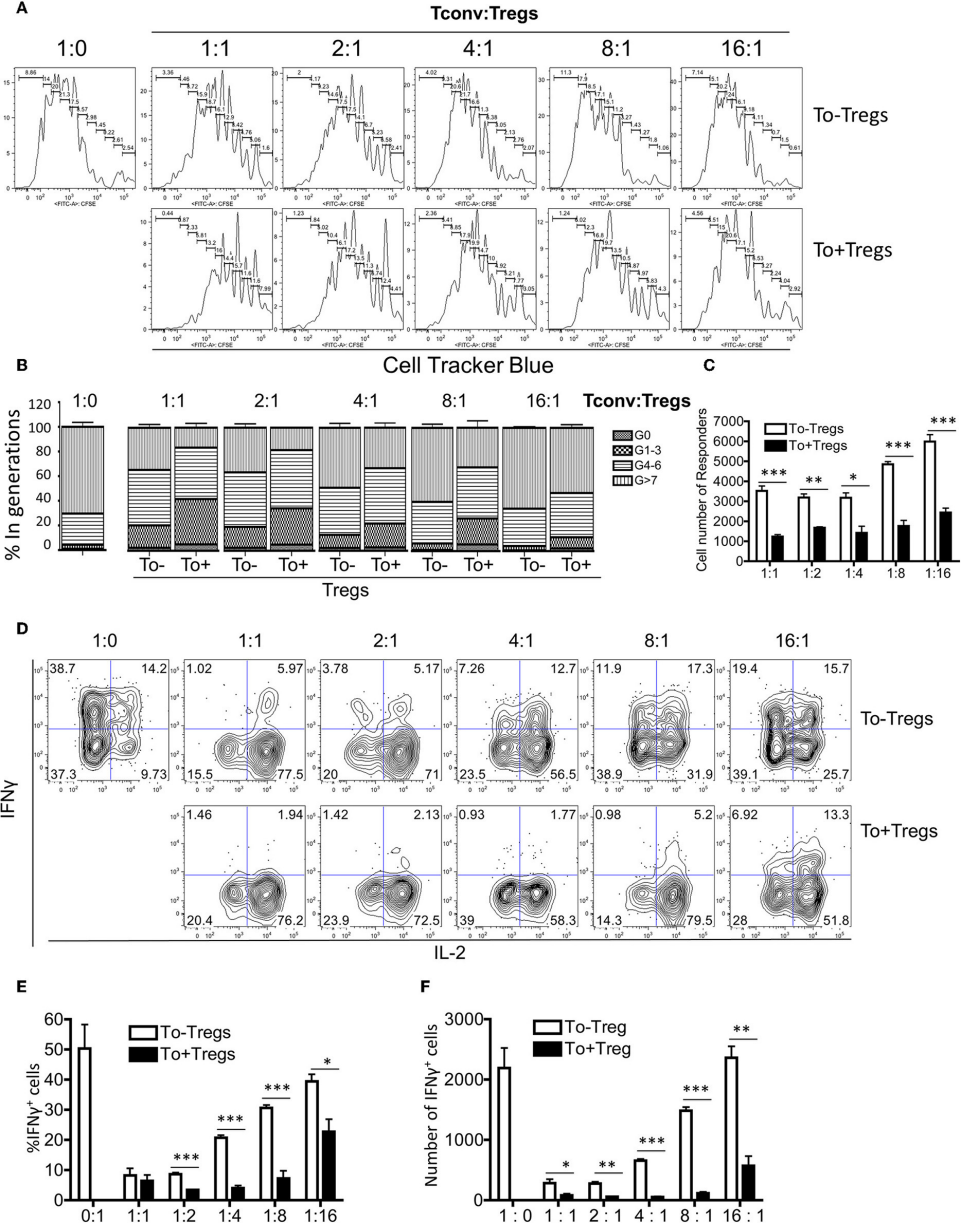
Functional analysis for eTregs. The tdTomato^−^ and tdTomato^+^ Tregs were sorted from the spleen of naïve mice. CD4^+^CD25^−^ T cells were labeled with CTB dye. Non-T cells were sorted and treated with mitomycin C as antigen presenting cells (APCs). In presence of APCs, soluble anti-CD3 and anti-CD28 antibodies, Tconv cells were co-cultured with either tdTomato^−^ or tdTomato^+^ Tregs at different ratios. The proliferation and cytokine production of Tconv cells were monitored. **(A)** Different suppressive effects of tdTomato^−^ and tdTomato^+^ Tregs on Tconv cell proliferation. **(B)** The quantification of Tconv cell generation progression for both groups. **(C)** The total Tconv cell number in both groups. **(D)** Representative plots of intracellular IFNγ and IL-2 expression in Tconv cells. **(E,F)** The percentage and total number of IFNγ+ Tconv cells in both groups. The data were collected from three independent experiments. *P < 0.05, **P < 0.01, and ***P < 0.001.

Tregs have been shown to undergo additional rounds of proliferation during homeostasis in comparison with conventional CD4 T cells (27). However, the Tlox method only resulted in labeling <10% of peripheral Tregs (Figures 1B,C). To determine whether eTregs represent a unique group or an arbitral marking of Tregs, we analyzed their transcriptional profile with a panel of Treg signature genes (28–30). The expression of well-established Treg signature genes including Foxp3, CTLA-4, and Foxo1 were comparable between eTregs and the rest of non-labeled Tregs (Figure 2A). However, most effector molecules, including LAG3, Granzyme B, Ebi3, TGFβ, IL-10, and IL-9, were significantly upregulated in eTregs (Figure 2B). To test whether eTregs are more potent effector suppressors, we co-cultured CellTracker™ Blue (CTB) labeled CD4^+^CD25^−^ Tconvs with either non-labeled Tregs or lineage tracked eTregs in the presence of antigen presenting cells (APCs) and soluble anti-CD3/anti-CD28 antibodies. The proliferation of Tconvs was measured by CTB dilution of generation progressions. We found that eTregs repressed the proliferation of Tconvs more effectively than the non-labeled Tregs (Figures 3A–C and Supplemental Table 1). Moreover, eTregs exhibited a stronger suppressive effect on Tconvs by inhibiting IFNγ production (Figures 3D–F). These observations indicated that eTregs are enriched with a group of highly potent effector Tregs, similar to the recently activated Tregs described in human studies (31, 32).

### Microbiota Drives the Generation of eTconvs and eTregs

**Figure 4:**
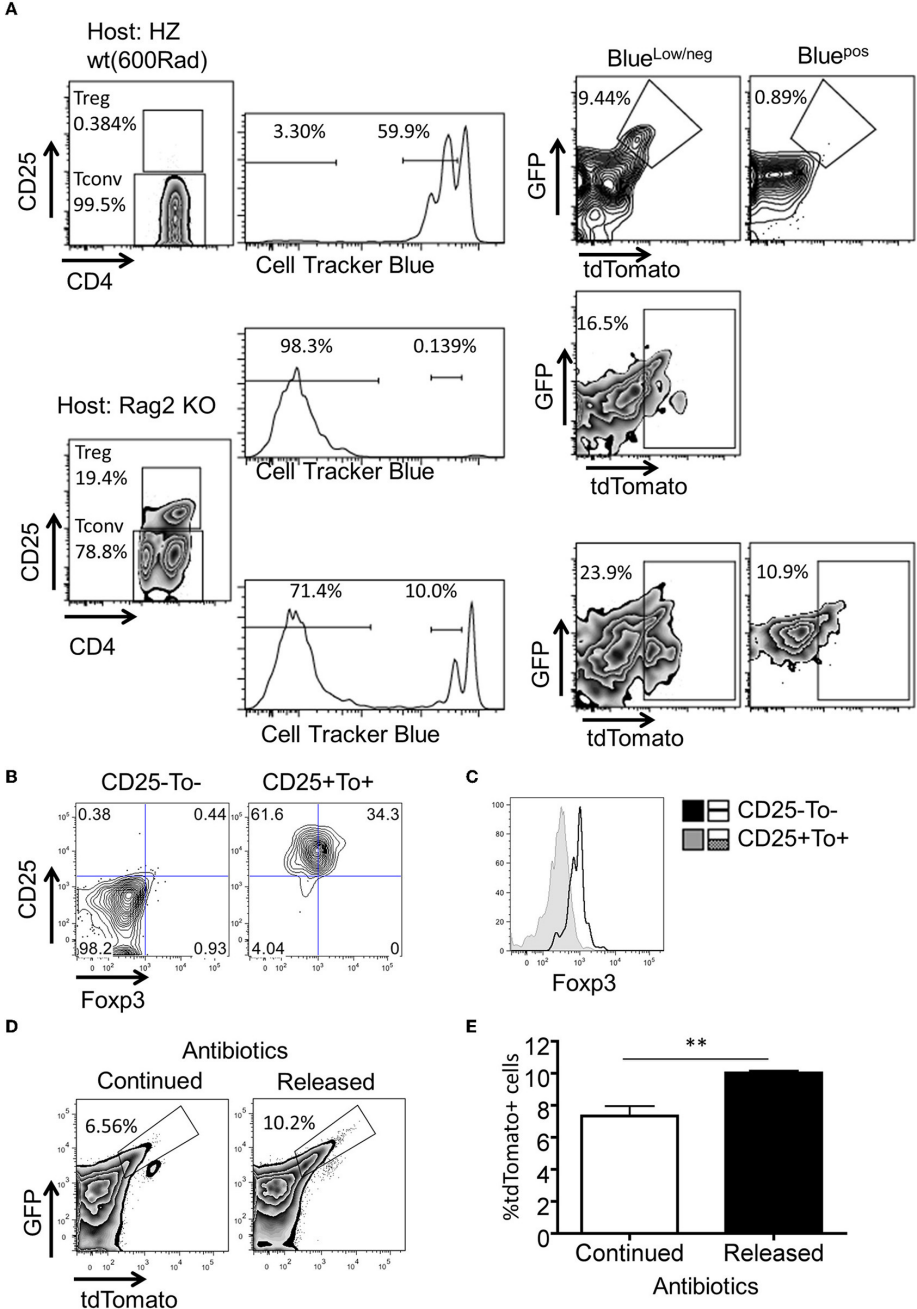
Microbiota antigens stimulate the expansion of eTregs. Purified tdTomato^−^CD4^+^ T cells were labeled with CTB dye and transferred to either sublethal irradiated WT mice or Rag2 KO mice. Four days post cell transfer, tdTomato expression in the splenocytes of the recipients was analyzed. **(A)** FACS plots for the percentage of tdTomato^+^ cells in subpopulations of donor cells. **(B,C)** FACS plots and histogram of Foxp3 expression in tdTomato^+^CD25^+^ cells from RAG2^−/−^ group. **(D)** The percentage of tdTomato^+^ Tregs in indicated groups. **(E)** Statistics for tdTomato^+^ cells in Tregs from two groups (*n* = 4). Each plot represents three independent animals. The experimental procedure for **(D,E)** was described in Methods. **P < 0.01.

It is not clear why the post proliferative eTregs only represent <10% of total Treg pool, even though all thymus derived Tregs are known to undergo proliferation before reaching to homeostasis in the periphery (27). We explored the possibility that Ox40Cre activity may not be even in all Tregs. We found that there is a low level expression of Ox40 in both Tconvs and Tregs regardless of tdTomato expression. When T cells get activation, Ox40 was highly expressed (Supplemental Figure 1C). Then we hypothesized that Ox40 induced Tlox activation may be associated with antigen driven clonal expansion but not homeostatic proliferation. In naïve animals, gut microbiota is the primary driving force of clonal expansion of Tconvs and generation of induced Tregs (19, 22, 33–35). To evaluate any possible role of microbiota in eTreg generation, we adoptively transferred CellTrack™ Blue labeled tdTomato^−^CD4^+^ T cells to Rag2 KO host, which is an established model for microbiota driven proliferation (36). As expected, donor T cells undergone extensive proliferation and resulted in the generation of Treg cells (Figures 4A–C). A significant fraction of these post expansion Tconvs and Tregs expressed tdTomato, indicating that microbiota can drive the generation of both eTconvs and eTregs from naïve CD4 T cells (Figure 4A, bottom panel). In contrast, the same donor CD4 T cells failed to activate the tdTomato marker when they undergone homeostatic proliferative in sublethal irradiated hosts (Figure 4A, top panel). The difference in activation of the tdTomato marker under these two experimental conditions cannot be explained by the difference in numbers of cell cycles. A direct comparison of Tconvs with equivalent low cycle numbers in both Rag2 hosts and sublethal irradiated hosts identified eTconvs in the former but not the latter. Therefore, Ox40Cre induced activation of the Tlox reporter in Tconv and Treg is fortuitously associated with microbiota driven expansion but not homeostatic proliferation. Of course, the part of expanded Treg cells could be converted from effector Tconv.

To further demonstrate that the proliferation of eTregs is indeed stimulated by microbiota antigens, we continuously treated nursing female mice by adding antibiotics in drinking water. This allowed us to deliver antibiotics to neonates through breast milk. After 4 weeks, mice were divided into two groups based on either continuing or terminating antibiotic treatment. The secession of antibiotic treatment allowed microbiota re-colonization in the gut. Interestingly, we found a significant increase of eTregs in microbiota re-colonizing group compared to non-stop treatment group (Figures 4D,E). Collectively, our data suggest that generation of eTregs is linked to clonal expansion in response to gut microbiota, but not to homeostatic proliferation.

### eTregs Are Clonally Related to Tconvs, but Not to the Non-lineage Tracked Tregs in the Peyer's Patch

To gain a better understanding on the origin of eTregs, we used MIDCIRS TCR-seq (37) to perform high-throughput sequencing of TCRβ after sorting tdTomato labeled or non-labeled Treg and Tconv fractions from spleen and Peyer's patch (PP). First, the rarefaction analysis on sequencing depth and saturation analysis on *Bhattacharyya* similarity index were performed to demonstrate that optimum sequencing depths were reached for *Bhattacharyya* similarity index analysis in all samples (Supplemental Figures 3A,B, Supplemental Tables 2, 3). CDR3 sequencing data from two independent mice showed that the length of CDR3 amino acid sequence as well as the distribution of TCRVβ usage were comparable among all sample groups from both spleen and PP (Supplemental Figures 4A–E), indicating there is no artificial bias during cell sorting and library construction regardless of the sample size variation between the labeled and non-labeled cells.

Based on the design principle of the Tlox system, we interpret TCR repertoire data based on the following premises: (1) All tdTomato labeled cells must have gone through clonal expansion. (2) tdTomato positive cells come from tdTomato negative cells but not vice versa. (3) Because the labeling frequency cannot be higher than 25% per cell cycle (25), both tdTomato positive and negative cells can be generated from the non-labeled founder cells. (4) Assuming Cre activity remains stable during clonal expansion, the frequency of non-labeled descendants reduces after each cell cycle, as such tdTomato labeled fractions will become the dominant population within the expanded clones.

**Figure 5:**
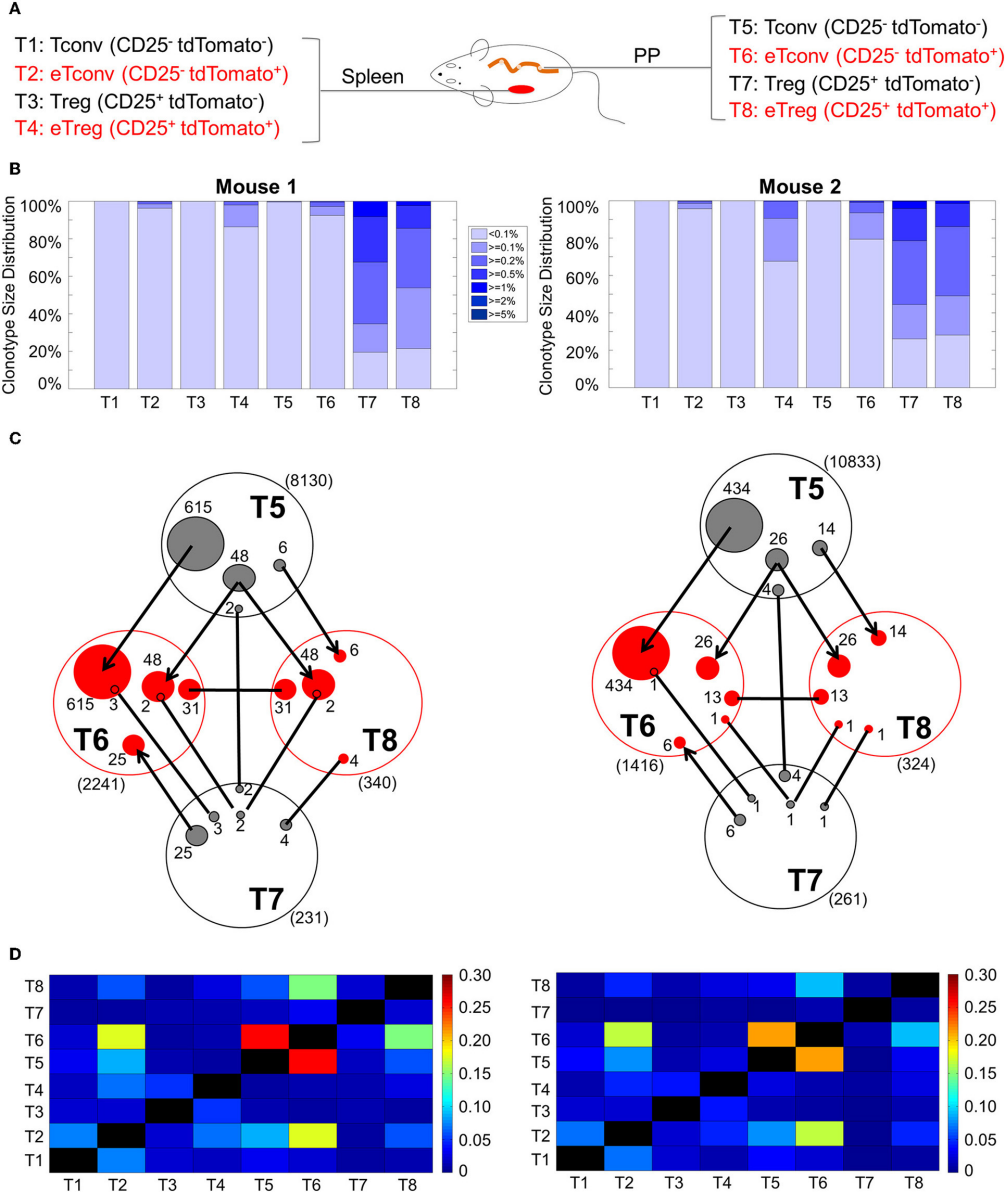
TCR clonal distribution and overlap TCRβ genes in CD4^+^ T cells. The Tconv cells and Tregs from both spleen and PP were separated into 4 populations as CD25^−^tdTomato^−^, CD25^−^tdTomato^+^, CD25^+^tdTomato^−^, and CD25^−^tdTomato^+^ cells. High throughout sequencing and analysis were described in Method. **(A)** The cell populations used for TCR repertoire sequencing. **(B)** Distribution of TCR repertoire clonotype size in all samples from two mice. The gradient color segments represent clonotypes which make up ≥5, ≥2, ≥1, ≥0.5, ≥0.2, ≥0.1, and <0.1% of total TCR repertoire. This distribution represents the extent of clonal expansion within each sample. **(C)** The sharing of unique clones between different populations from PP. **(D)** Comparison of TCR repertoire Bhattacharyya similarity index among different samples within each mouse. This index measures the similarity between two repertoires, which is based on the abundance of shared TCR sequences within these two populations.

Following the above principle, we predicted that tdTomato labeled cells are enriched of high frequency clones due to clonal expansion. An examination of clonal distributions indeed revealed signs of varying degrees of clonal expansion among all tdTomato labeled cell fractions (Figures 5A,B). Unexpectedly, the non-labeled Treg in PP showed a prominent and a similar pattern of clonal expansion as in eTregs, even though the former is 10 times larger than the latter in terms of the population size (Supplemental Figure 4A). To determine whether the two Treg populations defined by tdTomato labeling is clonally related or not, we compared nucleotide sequence identity between the two repertoires (Figure 5C). The total number of shared clones (defined by the combination of V gene segment, J gene segment and CDR3 nucleotide sequence) was found in a similar range between labeled (T8) and non-labeled (T7) Tregs within both mice. Only six clones in the first mouse and two in the second mouse were shared between the eTregs and the non-labeled Tregs. In contrast, the same analysis identified that 54 clones in the first mouse and 40 clones in the second mouse were shared between eTregs and non-labeled Tconvs (T5). The higher frequency of sharing with non-labeled Tconvs cannot be simply explained by the larger population size of Tconvs because sharing between these non-labeled Tconvs with non-labeled Tregs were only seven and five clones for the first and second mouse, respectively. Among the shared clones between eTregs and non-labeled Tconvs, majority of the clones were also shared with eTconvs (T6). Furthermore, we found 31 clones in mouse one and 13 in mouse two were uniquely shared between labeled eTregs and labeled eTconvs. The closer link between eTregs and Tconvs rather than with non-labeled Tregs indicates that eTregs are result of clonal activation and expansion starting from Tconvs. These clonal expansion events resulted in descendant clones located in both eTreg and eTconv compartments. Many of these lineage-converted Treg clones, defined by this repertoire analysis, were high frequency clones within the eTreg population (Figure 5C), indicating that eTregs have gone through extensive clonal expansion upon lineage conversion.

This analysis also revealed that the highest clonal sharing is between labeled and non-labeled Tconvs. The number of clones exclusively shared between non-labeled Tconvs (T5) and eTconvs (T6) was more than 10 times higher than all the eTreg (T8) clones that can be tracked back to non-labeled Tconvs (T5) (Figure 5C: 615 vs. 54 unique clones in the first mouse and 434 vs. 40 unique clones in the second mouse). This result indicates that <10% of activated Tconvs show lineage conversion to Tregs.

To further examine the relationship between PP T cells and circulating T cells in the spleen, we used the nucleotide sequence to calculate pairwise Bhattacharyya similarity for all individual samples obtained from spleen and PP (Figure 5D, Supplemental Figure 5, Supplemental Tables 3, 4). The highest similarities were found between the PP Tconvs and PP eTconvs pair followed by the PP eTconvs and Spleen eTconvs pair, and the PP eTconvs and PP eTregs pair. The similarity score between spleen eTconvs and spleen non-labeled Tconvs is much lower than that observed between PP eTconvs and PP non-labeled Tconvs. This finding supports the idea that most circulating eTconvs in the spleen are result of initial clonal activation taking place in the PP. Together, these data suggest that eTregs in the PP are the result of clonal activation of PP Tconvs. Most non-labeled Tregs in the PP may have a distinct origin different from that of eTregs.

### eTregs Express iTreg Markers and Could Be Induced From Tconvs *in vivo*

**Figure 6:**
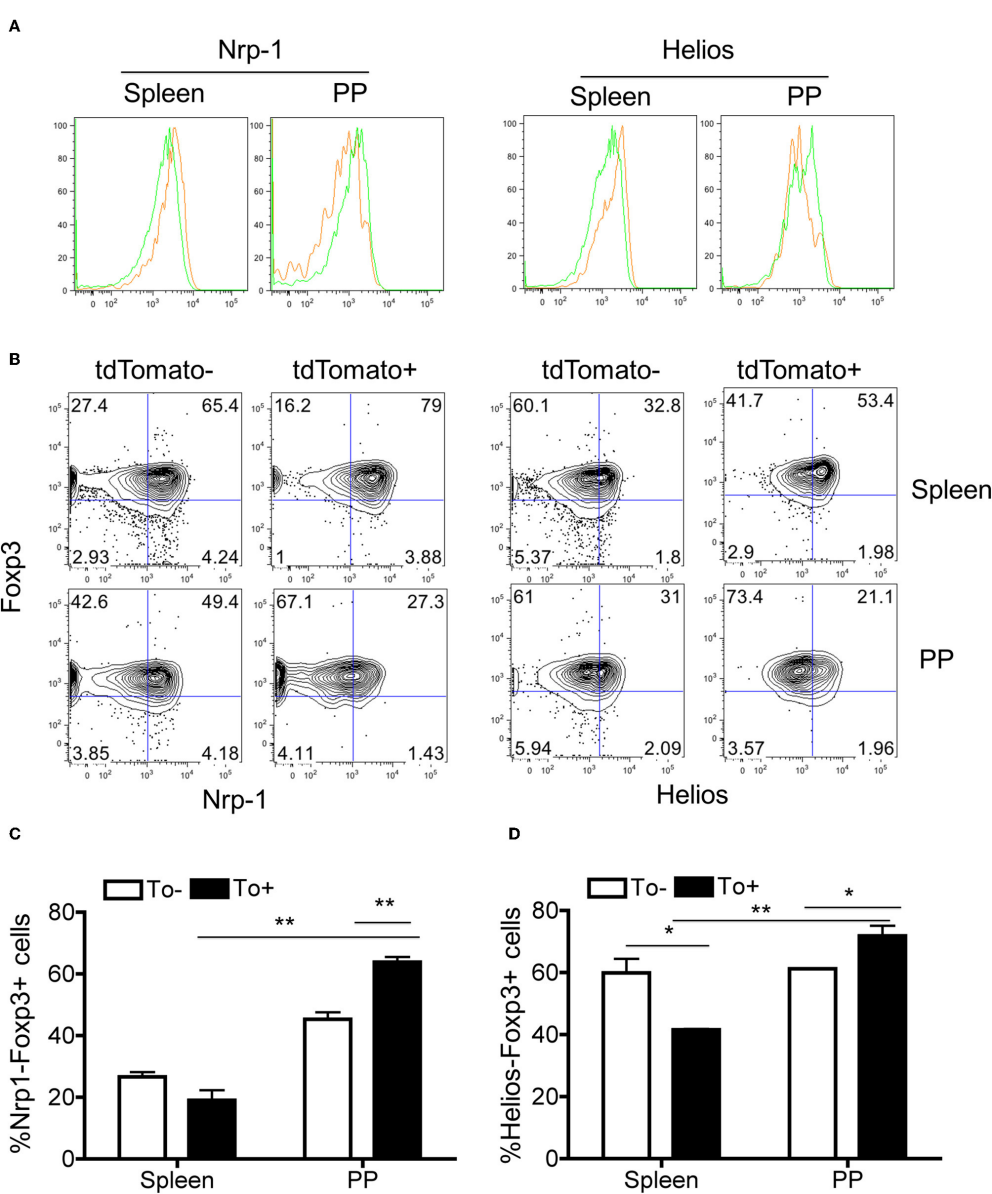
The expression of iTreg-associated genes in eTregs. Spleen and PP were harvested from Tlox/Ox40Cre mice. The Nrp-1 and Helios expressions in Tregs from both tissues were analyzed. **(A)** Histogram for Nrp-1 (Left) and Helios (Right) expression in tdTomato^−^ and tdTomato^+^ eTregs. The Green and orange line represents tdTomato^−^ and tdTomato^+^ cells. **(B)** The percentage of Nrp-1- cells (Left) and Helios- cells (Right) in tdTomato^−^ and tdTomato^+^ Tregs. **(C)** Quantification of Nrp-1-Foxp3^+^ cell percentage in tdTomato^−^ and tdTomato^+^ Tregs. **(D)** Quantification of Helios-Foxp3^+^ cell percentage in tdTomato^−^ and tdTomato^+^ Tregs. Each histogram and plot represent for three independent mice. *P < 0.05, **P < 0.01.

**Figure 7:**
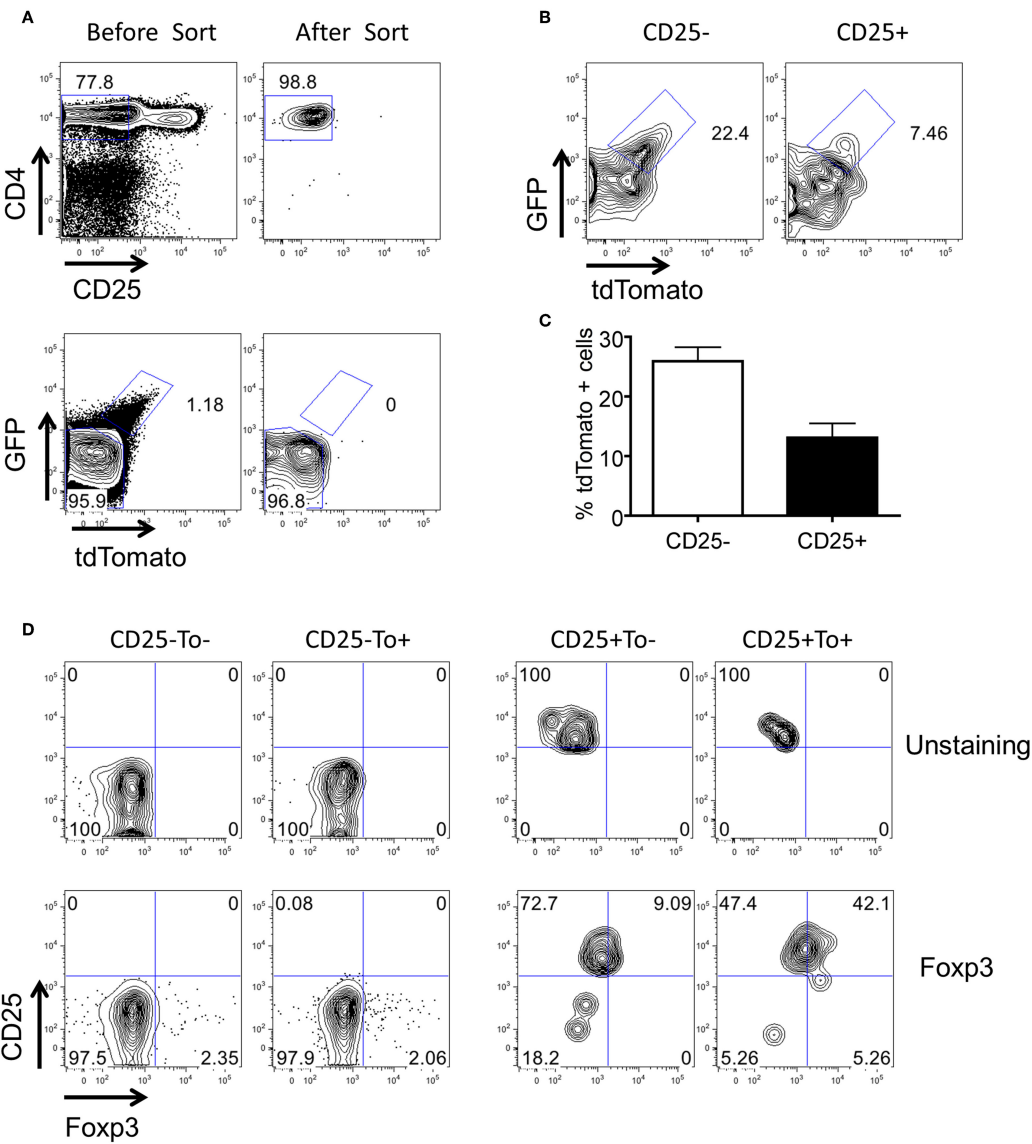
Conversion of tdTomato^−^ conventional CD4^+^ T cells to eTregs *in vivo*. 5 × 10^6^ CD4^+^CD25^−^tdTomato^−^ T cells were purified from the spleen of Tlox/Ox40Cre mice and adoptively transferred to lethal irradiated Th1.1+ hosts together with 1 × 10^7^ T cell-deleted Bone marrow cells. Three weeks later, donor cells were harvested from spleens of the hosts. The tdTomato and Foxp3 expression were analyzed in the donor cell population. **(A)** Sorting strategy of CD4^+^CD25^−^tdTomato^−^ donor T cells. **(B)** The tdTomato expression in the donor-derived CD4^+^CD25^−^ and CD4^+^CD25^+^ T cells. **(C)** Statistics of tdTomato expression in donor-derived CD4^+^CD25^−^ and CD4^+^CD25^+^ T cells. **(D)** FACS plots for foxp3 expression in the donor-derived CD25^−^ and CD25^+^ T cells. Data were collected from three independent mice.

Nrp-1 and Helios were previously reported as markers to distinguish between iTregs and nTregs (38–40). These two markers are expressed considerably lower in iTregs compared to nTregs. Both Nrp-1 and Helios were significantly downregulated in eTregs compared with non-labeled Tregs in PP but not in the spleen (Figures 6A–D). This observation further corroborates with the TCR repertoire analysis and supports the idea that PP eTregs is derived from activated Tconvs in the gut. Finally, we tested the ability of non-labeled Tconvs to give rise to tdTamato labeled T cells by transferring tdTomato^−^ Tconvs into lethal irradiated wild-type hosts (Figure 7A). tdTomato^+^ eTregs expressing foxp3 were detected together with tdTomato^+^ Tconvs 3 weeks post adoptive transfer (Figures 7B–D). Thus, eTregs could be directly induced from Tconvs that were forced to undergo microbial driven expansion in the lymphopenic hosts.

## Discussion

In this study, we used the Tlox recombination system to reveal the lineage relationship between post-expansion eTregs and effector T cells in the Peyer's patch. The Tlox system is dependent on Cre-mediated sister chromatid exchange that occur during cell cycles. We chose Ox40Cre to drive the recombination and the lineage marker activation because of its known activity in both activated T effector cells and Tregs. Nonetheless, this Ox40Cre transgene appears to selectively drive sister chromatid exchange in microbial-induced clonal expansion. It is apparently inefficient in driving sister chromatid exchange among thymic derived nTregs, which are known to undergo homeostatic proliferation after their initial formation in the thymus. While it is not clear whether the differential behavior of Ox40Cre transgene in homeostatic proliferation vs. microbial-induced clonal expansion is due to any difference in levels of Cre expression or other unknown attributes that affect the efficiency of sister chromatid exchange, the Ox40Cre-activated Tlox system, fortuitously, permitted us to label and study the T cell clones resulting from microbial driven clonal expansion in the PP.

Because the TCR repertoire between eTregs and the non-labeled Tregs are non-overlapping, we argue that eTregs captured by the Tlox labeling method represent a majority of microbial induced Tregs in the PP. Consistent with this view, we find a significantly greater number of eTreg clones than non-labeled Treg clones that are related to the non-labeled Tconv population according to their sequence identity, even though the population size of the labeled eTregs is only 1/10 of non-labeled Tregs. Therefore, we conclude that most non-labeled Tregs are unlikely derived from peripheral conversion from Tconvs, at least not through the same conversion mechanism that produces eTregs. Our result is consistent with the previous report based TCR repertoire analysis of a TCRβ transgenic line, which concluded that <10% of Tregs are peripherally converted from T effectors (41).

Our experimental system dictates that tdTomato labeled cells must come from a population of unlabeled cells. A closer examination of the non-labeled Tregs revealed a group of clones that share lineage identity with labeled eTconvs. Many of these are high frequency clones in the non-labeled Treg population, confirming that they have undergone certain degree of clonal expansion. Interestingly, the corresponding clones in the labeled eTconv population are invariably rare clones with most of them only appeared once in the sequenced pool. This cannot be easily explained by either sequencing error or contamination during cell sorting because we did not observe similar types of overlaps between non-labeled Tregs with other populations, such as the non-labeled Tconvs (which has much bigger population size than that of eTconvs). One possible interpretation is that these events are a result of clonal expansion of pre-existing nTregs, which led to acquisition of the tdTomato marker. It has been shown that forced activation and clonal expansion of Tregs in tissue culture will lead to loss of the Treg phenotype and apoptosis after several rounds of cell cycles (28, 42). This explains why we only see them in small numbers in the eTconv pool and rarely in the eTreg pool.

Our study indicated that majority of clonal expansion captured by the Tlox tracking method are initiated in the PP rather than in the spleen in naïve mice. Can this lineage tracking method reveal the frequency of lineage conversion from the activated T effectors in the PP? Assuming clonal activation and expansion starts from individual cells in the non-labeled Tconv pool. If a clonal expansion proceeds without lineage conversion, the clonal descendants may include only labeled eTconvs but not labeled eTregs. If a clonal expansion is coupled with lineage conversion, the clonal descendants will include both eTconvs and eTregs. Based on this concept, we compared the sharing frequency between non-labeled Tconvs with either eTconvs or eTregs and find that the former is more than 10 times higher than the latter. This result allows us to estimate that <10% of clonal activation and expansion of T effector cells in the PP results in lineage conversion to Tregs. It remains to be determined whether this frequency is a reflection of gut homeostasis in naïve animals or subject to change during acute or chronic disease situations.

In summary, the study presented here not only revealed the frequency of lineage conversion from microbial activated effector T cells to Tregs in naïve mice but also offered a new method for quantifying, tracking, and isolation of peripherally induced Tregs. This method can be easily combined with any genetic models or disease models for further understanding the mechanisms of Treg generation and their unique functions in response to microbial antigens.

## Methods

### Mice and Reagents

Tlox transgenic mice were generated as previously described (25) and backcrossed to B6 background for over 10 generations. The Ox40Cre strain was purchased from Jackson Lab. R26^tdTomato^ mice were gifted from Fan Wang's lab at Duke University. Animals were bred and maintained in the SPF facility managed by the Laboratory Animal Center of Xi'an Jiaotong University. All animal procedures were approved by the Animal Care Committee of Xi'an Jiaotong University and conformed to the Guide for the Care and Use of Laboratory Animals published by the US National Institutes of Health.

The antibodies used were as follows: APC/Cy7 anti-mouse TCRβ (H57-597), APC anti-mouse TCRβ (H57-597), PE/Cy7 anti-mouse TCRβ (H57-597), APC/Cy7 anti-mouse CD4 (GK1.5), PE/Cy7 anti-mouse/human CD44 (IM7), PE/Cy5 anti-mouse CD25 (PC61), PE/Cy7 anti-mouse IFNγ (XMG1.2), APC anti-mouse IL-2 (JES6-5H4), Pacific Blue™ anti-mouse FOXP3 (MF-14), APC anti-mouse IL-17A (TC11-18H10.1), APC anti-mouse CD304 (3E12), Brilliant Violet 421™ anti-mouse/human KLRG1 (2F1/KLRG1), PE/Cy7 anti-mouse CD127 (A7R34), Biotin anti-mouse CD122 (TM-beta1), and PE/Cy7 Streptavidin were purchased from Biolegend. APC anti-mouse/rat Foxp3 (FJK-16s), PE anti-mouse/human helios (22F6), and Foxp3/Transcription Factor Fixation/Permeabilization Concentrate and Diluent were purchased from eBioscience. The APC BrdU Flow Kit was from BD Biosciences. All the antibiotics were purchased from Sigma.

### FACS Analysis

Single-cell suspensions were prepared from spleen, peripheral lymph nodes and Peyer's patches (PP), and stained with anti-TCRβ, CD4, CD25, CD44, and CD62L Abs in the dark at 4°C for 30 min. GFP and tdTomato expression were analyzed in Tconvs and Tregs with the FACSCanto II flow cytometer (BD Biosciences). Flowjo software (Tree Star) was used for data analysis.

To analyze intracellular transcriptional factors, tdTomato^+^CD25^−^ and tdTomato^+^CD25^+^ T cells were sorted with FACSAria (BD Biosciences) sorter. Purified cells were fixed and permeabilized according to the manual of Foxp3 kit, followed by anti-foxp3 and anti-Helios antibody staining and FACS analysis. In some experiment, Nrp-1 was stained before fixation.

For cytokine analysis, lymphocytes from spleen and PP were stimulated with PMA/Ionomycin in presence of Brefeldin A and monensin for 4 h *in vitro*. Cells were washed and stained with anti-CD4, CD25, and TCRβ antibodies. After 30-min incubation, cells were fixed and permeabilized according to BD Cytofix/Cytoperm™ Fixation/Permeabilization Kit, followed by IFNγ and IL-17 analysis with FACS.

### Gene Expression Analysis by Real-Time PCR

The tdTomato^−^ and tdTomato^+^CD25^+^ T cells were sorted by FACS, and followed by RNA extraction and cDNA synthesis with RNAqueous micro kit (Life Technologies) and M-MLV reverse transcriptase (Life Technologies), respectively. SYBR-based real-time PCR was done to determine relative gene expression.

### Adoptive Transfer of T Cells

In some experiment (Figure 4), tdTomato^−^CD4^+^ T cells from Tlox/Ox40Cre mice were labeled with CellTracker violet dye. 1 × 10^6^ labeled CD4^+^ T cells were adoptively transferred to either sublethal-irradiated (600 Rad) WT mice or Rag2 KO mice. Four days later, donor cells were recovered from spleen of host by Thy1.2 marker. The tdTomato expression was analyzed in CD25^−^ and CD25^+^ T cells.

In some experiment (Figure 7), CD4^+^CD25^−^tdTomato^−^ T cells were transferred to lethal irritated mice. Three weeks later, tdTomato and foxp3 expression were analyzed in donor cells.

### Mouse TCRβ Sequencing Library Generation

Total RNA from indicated cell populations was used for reverse transcription. Second strand synthesis using Superscript III (Life Technology) was done following manufacturer's suggested concentrations. Molecular barcodes were added during second strand synthesis. The illumina adaptors with indexes were added during second PCR making the final libraries. Libraries were gel purified, qPCR quantified and sequenced on Illumina Mi-seq with paired-end 250 bp read. More details can be found in Supplemental Table 5.

### Sequencing Data Processing and Analysis

Raw reads from Illumina MiSeq PE250 were first filtered, only reads have the exact corresponding sample's library index and retain TCR constant sequence were kept for further analysis. These reads were then cut to 150 nt starting from constant region to eliminate high error-rate prone region at the end of reads, and split into MID (Molecule Identifiers) groups based on 12 nt barcoded sequences. From each MID group of reads, one single consensus sequence was generated based on the consensus of nucleotides weighted by the quality score at each position. Following this method, each MID group or consensus sequence is equivalent to one RNA molecule. These RNA molecule sequences were used for further analysis.

In order to compare the similarity between different samples, Bhattacharyya similarity index (43, 44) based on the shared clonotypes between repertoires were adopted. The value of Bhattacharyya similarity index ranges from 0 to 1, with 0 means no overlap between two repertoires while 1 means two identical repertoires. MIGEC (45) tool was used for CDR3 annotation and V/J gene segments assignment.

### Antibiotics Treatment

The feeding female mice were administrated with the cocktail of antibiotics (0.5 mg/ml Wancomycin HCL, 0.66 mg/ml Ciprofloxacin and 2.5 mg/ml Metronidazole in 20 mg/ml filtered sugar-sweetened grape Kool-Aid Mix water) through drinking water. The neonates at Day 1 post birth take in the antibiotics through the milk for 4 weeks till weaning age. Then, the litter was divided into two groups. One group of mice was continually fed with the same cocktail of antibiotics through water, while the other group of mice was fed with sugar-sweetened water as control. One week later, lymphocytes from spleen were harvested, and tdTomato expression in CD4^+^CD25^+^ T cells was analyzed with FACS.

### *In vitro* Suppressive Assay for Tregs

In presence of 1 × 10^4^ mytomycin C treated TCRβ- splenocytes, 0.5 μg/ml soluble anti-CD3 and 1 μg/ml anti-CD28 Abs, CellTracker violet dye (CVD) labeled 1 × 10^4^ CD4^+^CD25^−^ T cells (Responders) were cocultured with different ratio of tdTomato^−^ or tdTomato^+^ CD4^+^CD25^+^ T cells (Tregs) for 3 days. CVD dilution was analyzed using FACS, and the percentage in each generation and total number of responders were calculated. In some experiment, cells from the above culture were stimulated with PMA/Ionomycin in presence of Brefeldin A/Monessin for 4 h, and IFNγ expression in responders was analyzed using FACS.

## Data Availability Statement

The raw data supporting the conclusions of this article will be made available by the authors, without undue reservation, to any qualified researcher.

## Ethics Statement

The animal study was reviewed and approved by the Animal Care Committee of Xi'an Jiaotong University.

## Author Contributions

KZ, S-QL, XY, and LL performed mouse model and *in vivo* experiment. XZ, LS, DZ, TX, and YC performed *in vitro* culture. TX, YS, HZ, and XS performed FACS analysis and sorting. CH, MQ, and NJ performed TCR analysis. QL, NJ, and BZ performed experimental design and writing.

### Conflict of Interest

NJ is a Scientific Advisor of and holds equity interest in ImmuDX, LLC and Immune Arch, Inc., two startups that are developing products related to the research reported here. CH is a consultant of ImmuDX, LLC and Immune Arch, Inc. The remaining authors declare that the research was conducted in the absence of any commercial or financial relationships that could be construed as a potential conflict of interest.

## Supplementary Material

Supplemental Figure 1 The tdTomato expression in CD4^+^ T cells from R26^tdTomato^/Ox40Cre mice. R26^tdTomato^ mice were crossed with Ox40Cre transgenic mice. The tdTomato expression was analyzed in CD4^+^CD25^−^ and CD4^+^CD25^+^ T cells. **(A)** FACS plots for tdTomato expression in CD4^+^CD25^−^ and CD4^+^CD25^+^ T cells from thymus and LNs. **(B)** Quantification of tdTomato^+^ cell percentage in CD25^−^ and CD25^+^ T cells (*n* = 3). **(C)** Ox40 expression in the indicated cell populations.

Supplemental Figure 2 Phenotype of tdTomato^+^ conventional T cells from Tlox/Ox40Cre mice. **(A)** FACS plots for CD44 and CD62L expression in tdTomato^−^ and tdTomato^+^ CD4^+^CD25^−^ T cells. **(B)** Statistics of tdTomato^+^ cells in CD4^+^CD25^−^ T cells (Left), and CD44^+^CD62L^−^ cell percentage in tdTomato^−^ and tdTomato^+^ conventional T cells (Right). **(C)** Representative FACS plots of tdTomato^+^ cells in Tconv and Tregs from different lymph organs. **(D)** Statistics for **(C)**. The data is represent of three independent mice.

Supplemental Figure 3 Saturation analysis of sequencing depth. **(A)** Rarefaction analysis of clonotype number. In order to evaluate whether sequencing depth was saturated or not, rarefaction analysis was performed. Random reads of increasing amount were subsampled from the raw reads dataset, and the numbers of clonotypes were calculated from each set of subsampled reads. Similar analysis was done for both mice. This representative figure was from mouse 1. The inside figure is for sample M1T7 and M1T8. **(B)** Rarefaction Analysis on Bhattacharyya Similarity Index. Rarefaction analysis was used to study the relationship between sampling depth and Bhattacharyya similarity index estimation (46). Subsampling was performed on a level of RNA molecules. Hundred percentage corresponded to all the RNA molecules obtained at the highest sequencing depth for each sample. Increasing percentage of RNA molecules was randomly subsampled from both target RNA molecule datasets, then similarity index between the two subsets were calculated. The similarity index increases with increasing subsampling depth initially then reaches a plateau. The dash line represents the sampling depth we used to calculate similarity index. This is one representative of two mice.

Supplemental Figure 4 TCR repertoire coverage and V gene segment usage analysis for conventional and regulatory T cells. **(A)** Summary of diversity coverage in all repertoires. The diversity coverage is calculated as the number of unique clonotype divided by the number of cells. Clonotype is defined on different levels: unique RNA sequence and unique CDR3 nucleotide sequence (A combination of V and J segments at nucleotide level). **(B,C)** CDR3 Amino Acid length distribution for repertoires within each mouse. These two figures show no significant difference. **(D,E)** Frequencies of V beta gene segment usage within all the samples of each mouse.

Supplemental Figure 5 Clonal frequency of shared clones among different fraction of T cells in the Peyer's patch. Pie charts illustrate clonal frequencies of shared clones between indicated populations. Major populations that are shared between different cell fractions are labeled in the corresponding slices and are indicated with the same color. The frequencies of non-overlapping clones are shown in the gray slices. The population size for each cell fraction is indicated in the parentheses underneath each pie chart.

Supplemental Table 1 Percentage of conventional T cells in each divided generations. The experiment was described in Method and Figure 3. The average percentage of Tcon cells in each generation was shown in the table. ^*^*P* <0.05, ^**^*P* <0.01, and ^***^*P* <0.001. This data is representative of three independent experiments.

Supplemental Table 2 Similarity index variance estimation based on bootstrap. Bootstrap method was introduced to estimate the similarity index variance. Similarity index was calculated from each bootstrapped sample, which is randomly resampled (with replacement) from total RNA molecules until reach the same size of the original dataset. On average, ~60% of distinct RNA molecules in the original dataset will be covered in each new sample (47). After repeating this procedure for 100 times, mean and standard deviation were estimated.

Supplemental Table 3 Bhattacharyya similarity index between different samples. The similarity between 16 samples from two mice was compared by calculating Bhattacharyya similarity index. The value of similarity index between all pairs was shown in the table. The highlight indicated the similarity index within the same animal.

Supplemental Table 4 Summary of the overlapping clones among different populations from Peyer's patch. The absolute overlapping clone number was shown between two, three, and four different populations. The percentage of shared clone number in each group was calculated. The data presents two independent mice.

Supplemental Table 5 Primers used for PCR amplification of TCR cDNA library preparation. The RT primer was used for reverse transcription and the second strand primer was used for second strand synthesis. The 1st PCR forward primer and 1st PCR reverse primer were used for 1st PCR, and the 2nd PCR forward primer and 2nd PCR reverse primer were used for 2nd PCR. The 12 “N” in Second strand primer is the molecular identifier. The 6 “X” in 2nd PCR forward primer is library index for multiple sample pooling.
