# Supplementary material for: Lineage Tracking the Generation of T Regulatory Cells From Microbial Activated T Effector Cells in Naïve Mice
Source: Front Immunol. 2020 Jan 17;10:3109. doi: 10.3389/fimmu.2019.03109 (PMC6978744; doi:10.3389/fimmu.2019.03109)
Supplement: Supplemental Figure 1 — The tdTomato expression in CD4+ T cells from R26tdTomato/Ox40Cre mice. R26tdTomato mice were crossed with Ox40Cre transgenic mice. The tdTomato expression was analyzed in CD4+CD25− and CD4+CD25+ T cells. (A) FACS plots for tdTomato expression in CD4+CD25− and CD4+CD25+ T cells from thymus and LNs. (B) Quantification of tdTomato+ cell percentage in CD25− and CD25+ T cells (n = 3). (C) Ox40 expression in the indicated cell populations. [file Data_Sheet_1.PDF]

Supplemental Fig.1

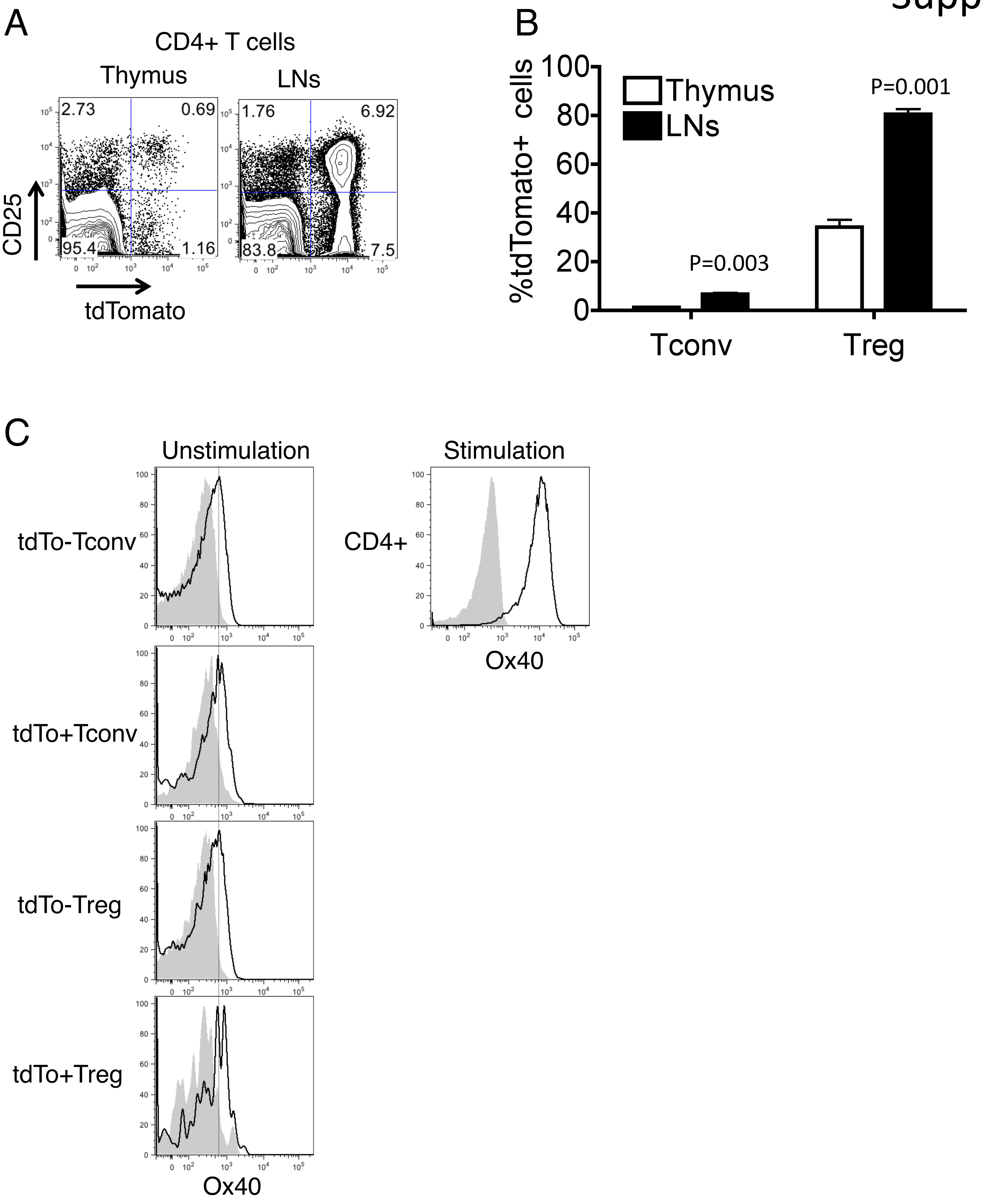

Supplemental Fig. 2

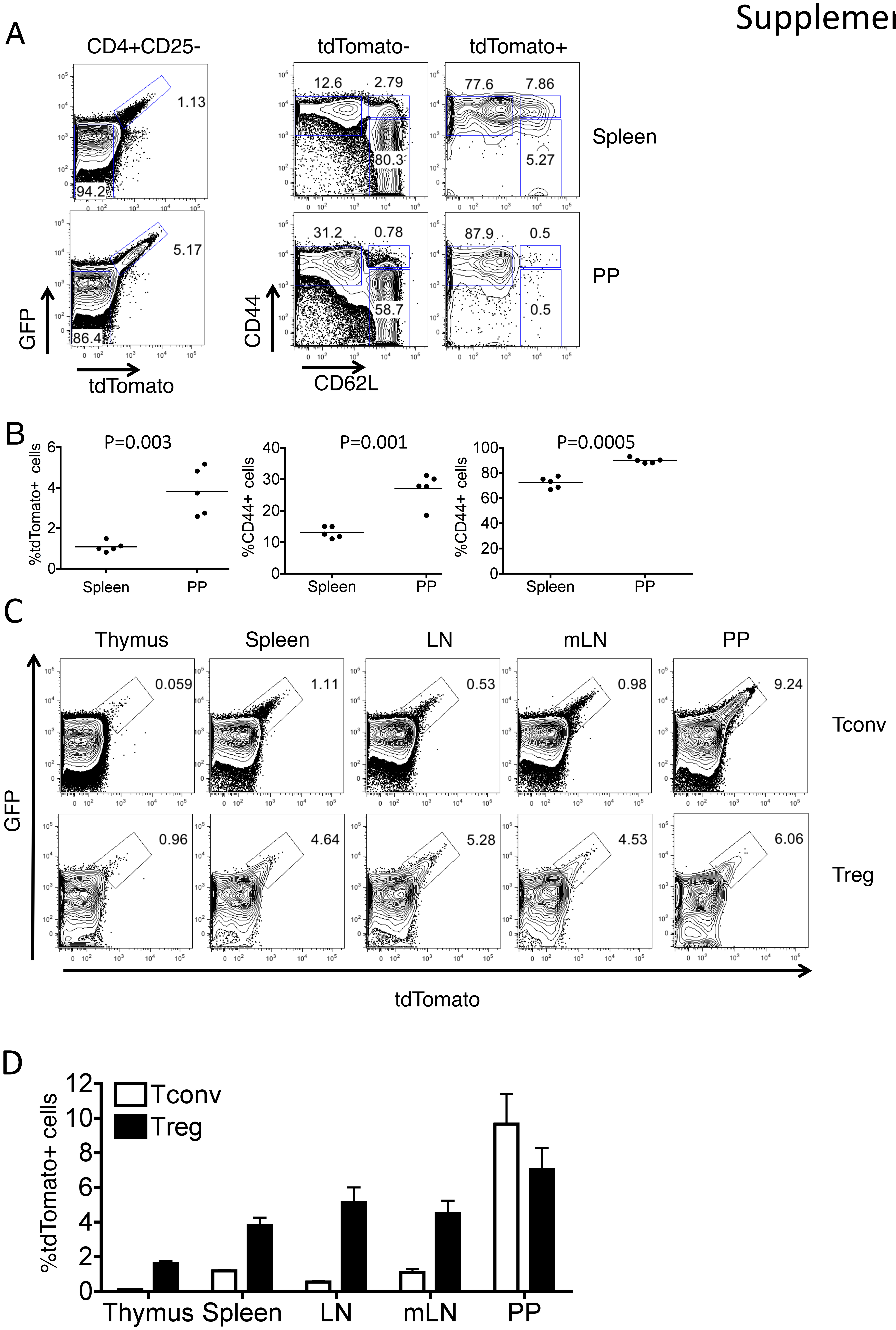

Supplemental Fig. 3

A

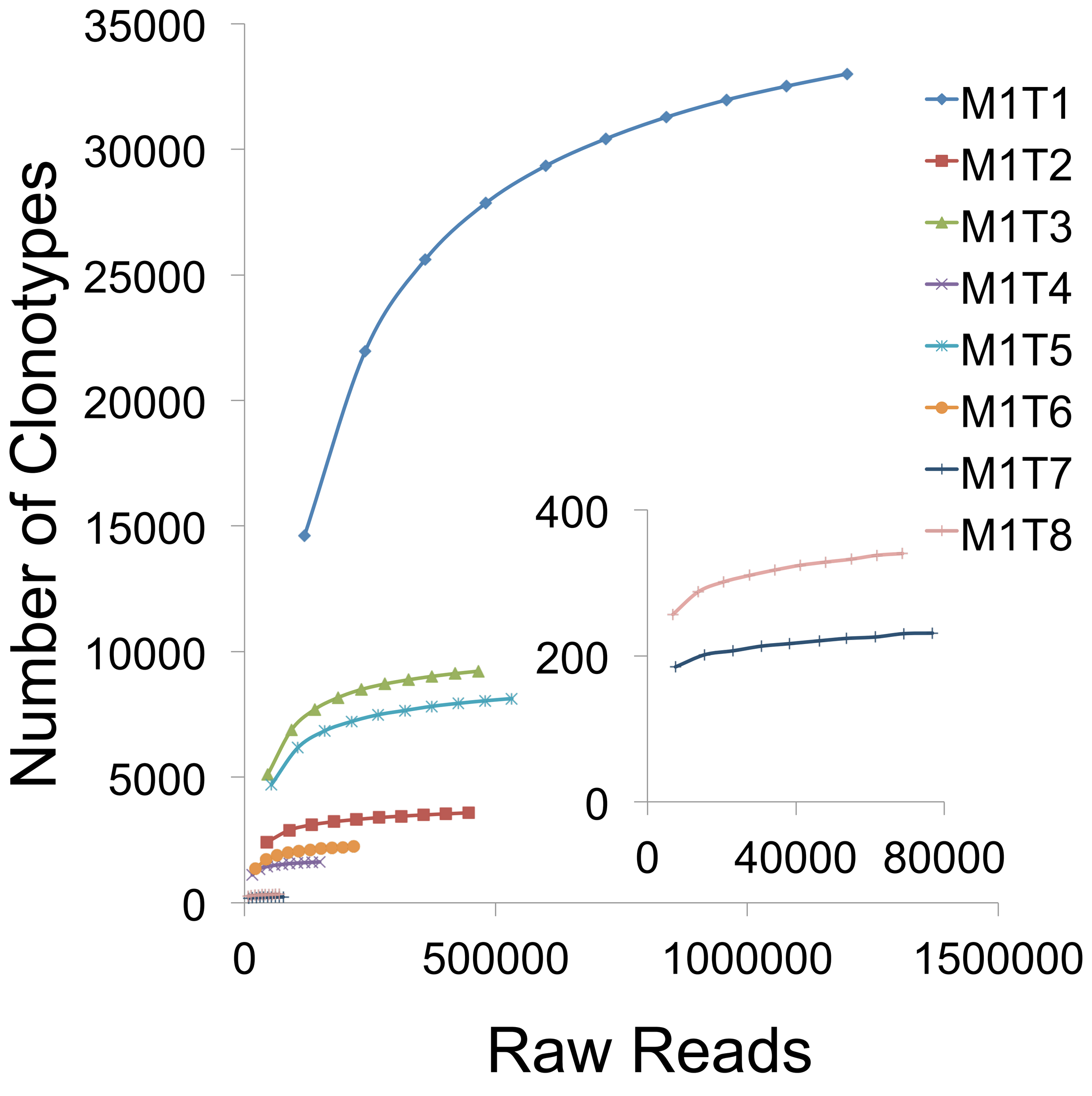

B

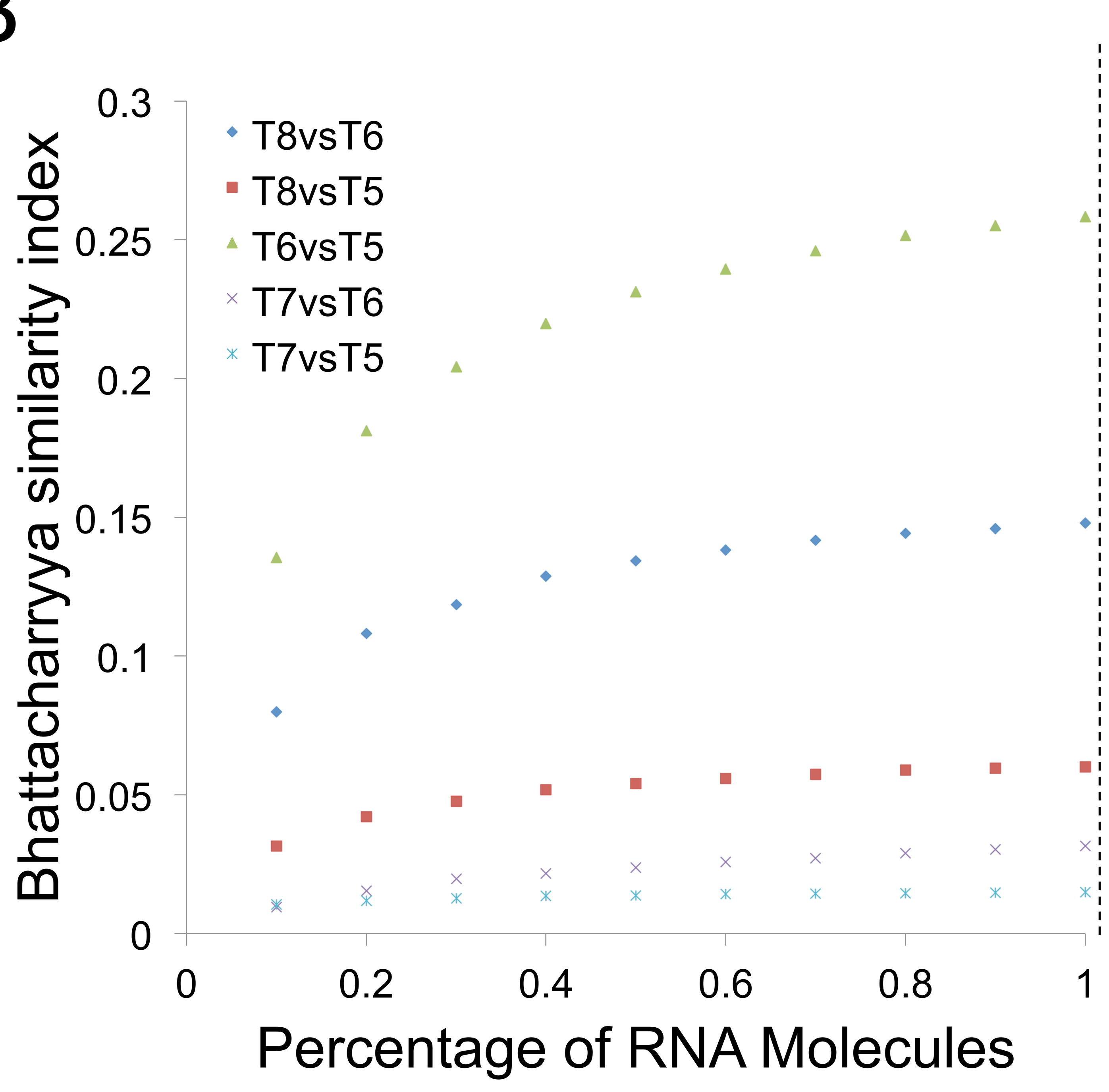

A

Samples				#Cell	Percentage of RNA input(%)	Diversity (unique_RNA_mol, 150bp)	Diversity (V, J CDR3 NT level)
M1T1	CD25-	Tomato-	Spleen	500000	0.05	38586	32997
M1T2	CD25-	Tomato+	Spleen	81400	0.05	4835	3581
M1T3	CD25+	Tomato-	Spleen	199900	0.05	10776	9221
M1T4	CD25+	Tomato+	Spleen	25000	0.05	2130	1627
M1T5	CD25-	Tomato-	PP	273900	0.05	9939	8130
M1T6	CD25-	Tomato+	PP	46500	0.05	3003	2241
M1T7	CD25+	Tomato-	PP	15461	0.05	331	231
M1T8	CD25+	Tomato+	PP	1726	0.25	505	340
M2T1	CD25-	Tomato-	Spleen	500000	0.05	31268	26364
M2T2	CD25-	Tomato+	Spleen	75500	0.05	3827	2732
M2T3	CD25+	Tomato-	Spleen	182199	0.05	9085	7523
M2T4	CD25+	Tomato+	Spleen	26364	0.05	1432	1071
M2T5	CD25-	Tomato-	PP	255900	0.05	13669	10833
M2T6	CD25-	Tomato+	PP	35390	0.05	2003	1416
M2T7	CD25+	Tomato-	PP	13720	0.05	347	261
M2T8	CD25+	Tomato+	PP	1480	0.25	436	324

B

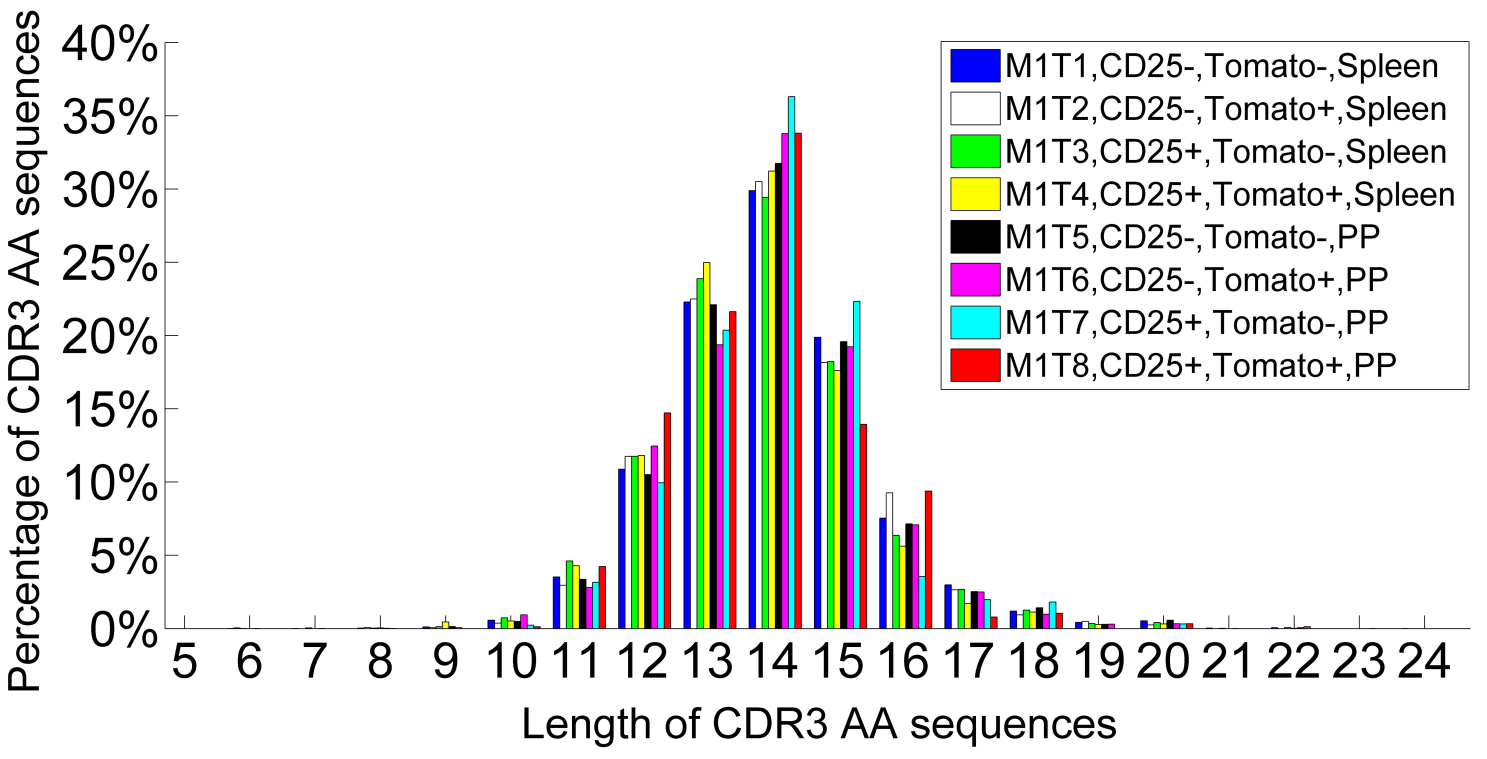

C

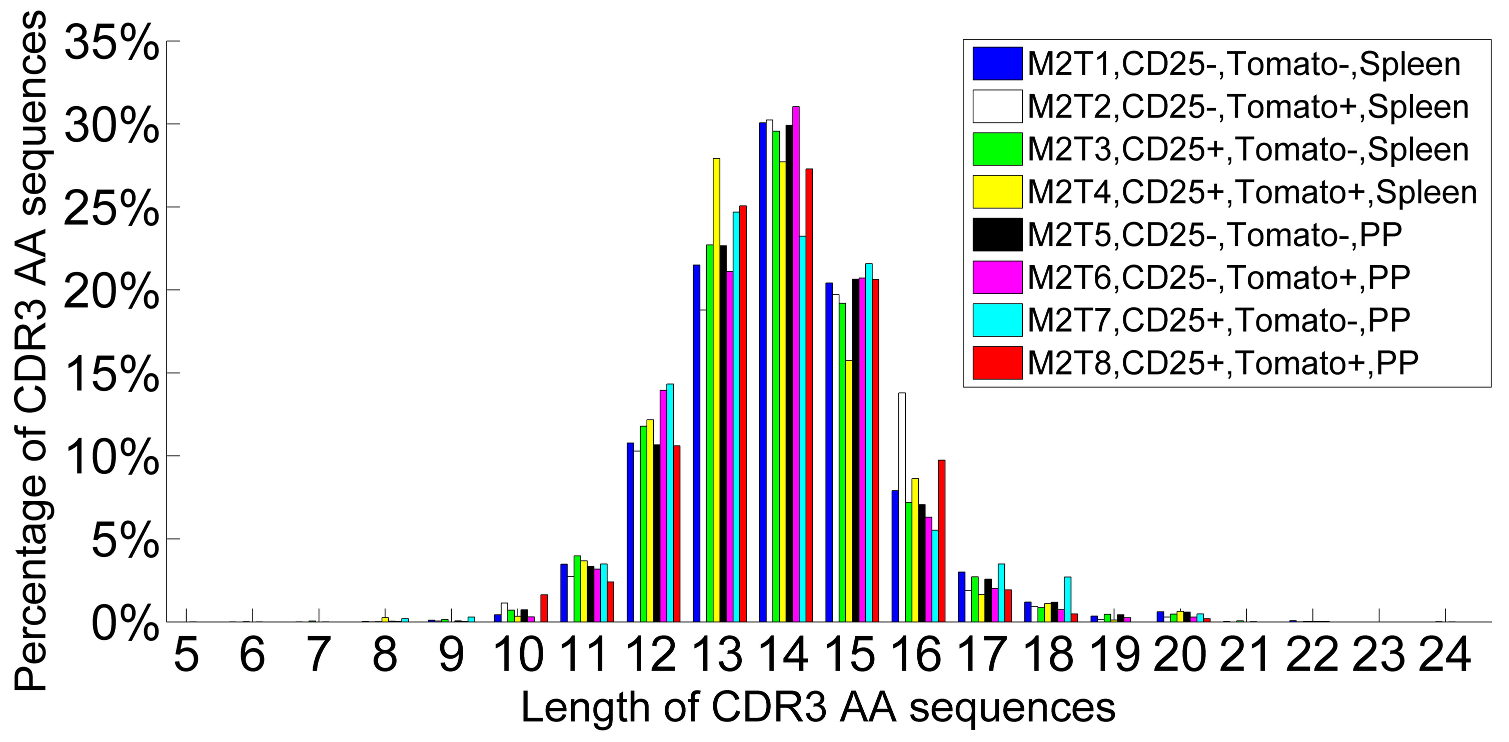

D

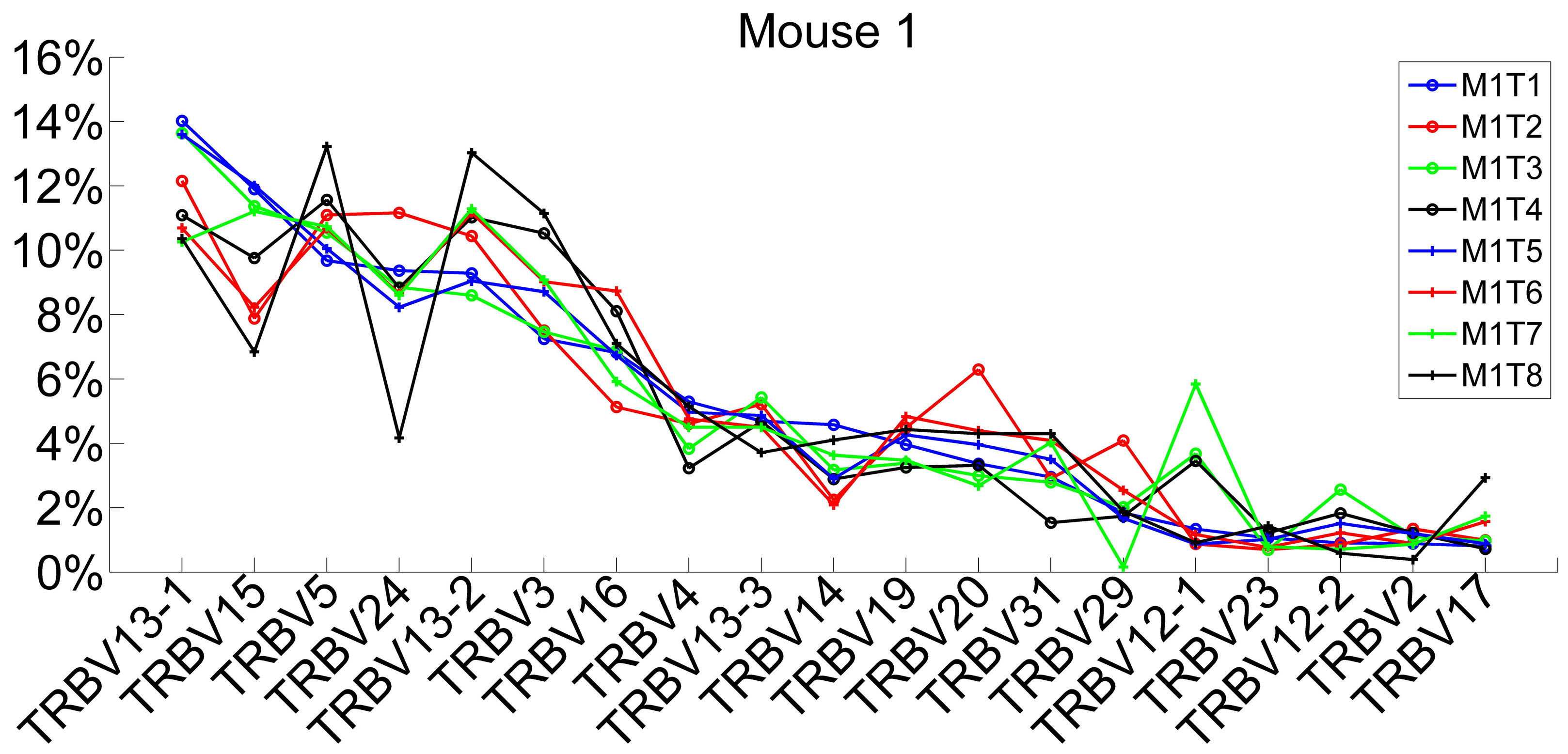

E

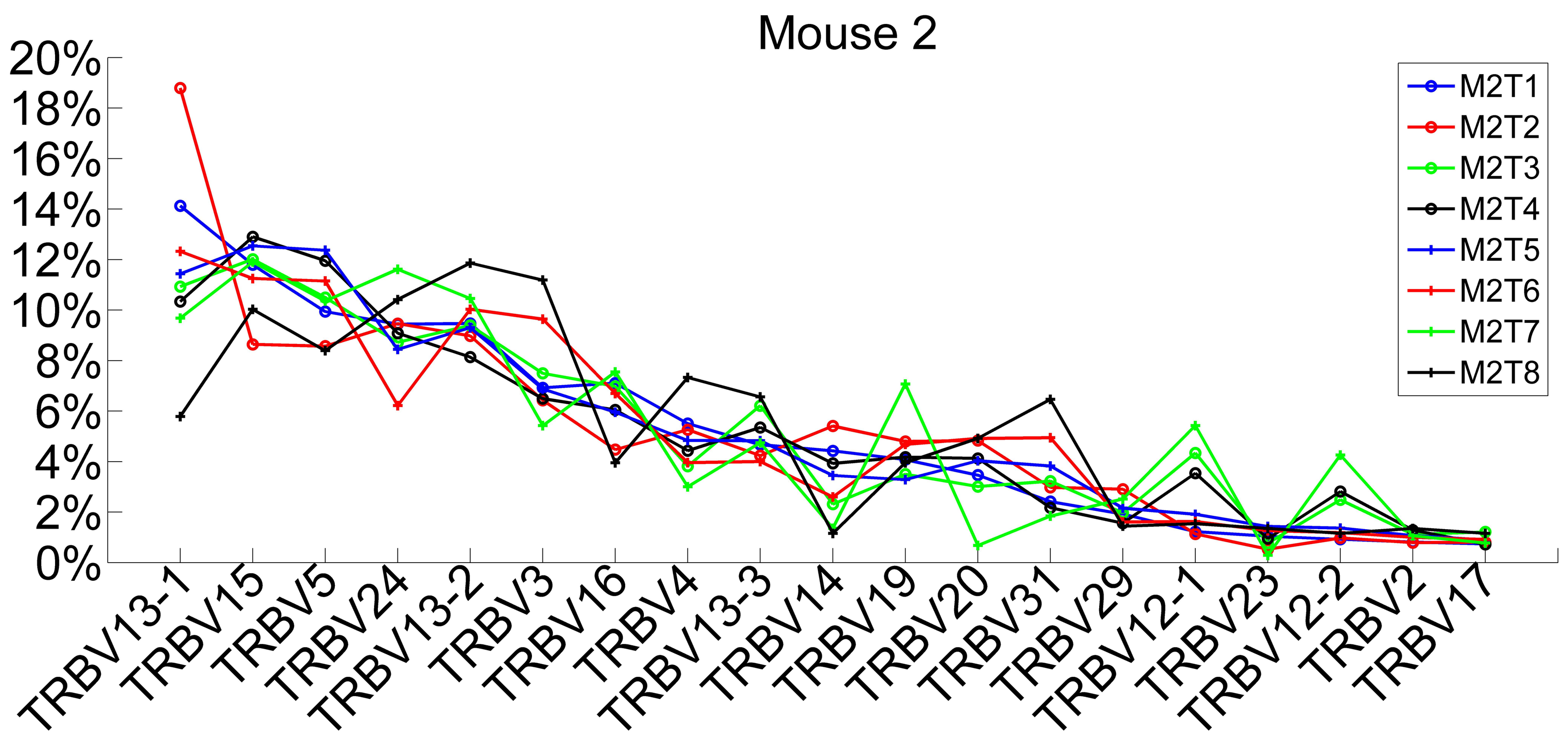

Supplemental Fig. 5

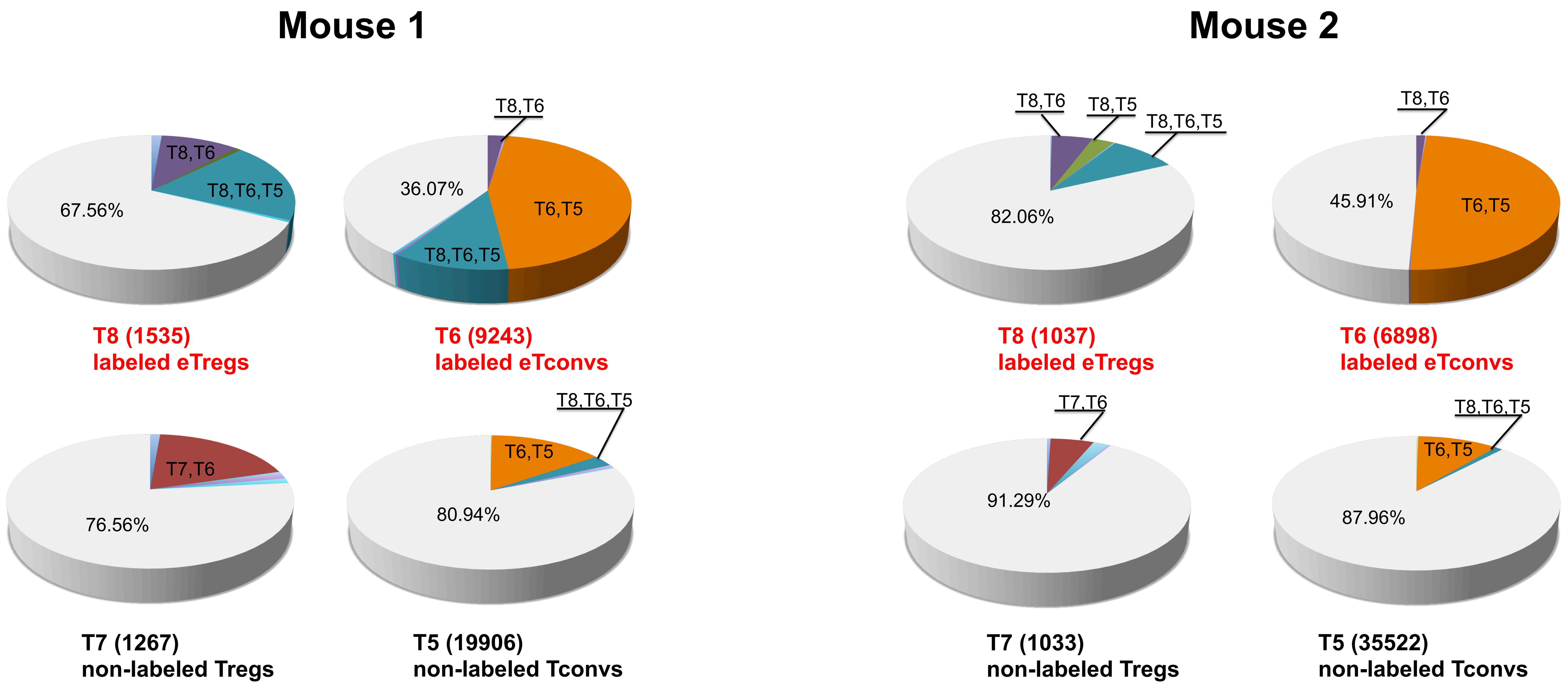

Supplemental Table.1 Percentage of conventional T cells in each divided generations

	G0		G1-3		G4-6		G>7	
	To-Tregs	To+Tregs	To-Tregs	To+Tregs	To-Tregs	To+Tregs	To-Tregs	To+Tregs
0 : 1	1%	2.27%*	3.35%	4.86%	25.26%	29.66%	70.44%	63.23%
1 : 1	2.41%	5.30%	18.26%	36.75%**	45.03%	41.78%	34.26%	16.22%*
1 : 2	2.45%	5.08%**	17.01%	29.19%**	44.26%	47.43%	36.31%	18.14%**
1 : 4	2.32%	2.78%	10.68%	19.38%*	38.23%	44.98%*	48.72%	32.86%*
1 : 8	0.95%	5.49%**	5.26%	20.96%*	33.78%	41.54%	59.98%	32.07%**
1 : 16	0.65%	2.25%*	3.52%	8.98%***	30.25%	35.88%	65.55%	52.8%**

Supplemental Table.2 Similarity index variance estimation based on bootstrap

Bootstrap	T8vsT6	T8vsT5	T6vsT5	T7vsT6	T7vsT5
**MEAN**	0.137754	0.055986	0.241947	0.030404	0.014251
**STDEV**	0.004519	0.002291	0.002265	0.001144	0.001517

Supplemental Table.3 Bhattacharryya similarity index between different samples

	M1T1	M1T2	M1T3	M1T4	M1T5	M1T6	M1T7	M1T8	M2T1	M2T2	M2T3	M2T4	M2T5	M2T6	M2T7	M2T8
**M1T1**	NA	0.0748	0.0222	0.0166	0.0306	0.0232	0.0080	0.0095	0.0260	0.0119	0.0139	0.0088	0.0166	0.0090	0.0009	0.0045
**M1T2**	0.0748	NA	0.0198	0.0692	0.0859	0.1744	0.0093	0.0571	0.0122	0.0106	0.0090	0.0070	0.0094	0.0073	0.0021	0.0048
**M1T3**	0.0222	0.0198	NA	0.0483	0.0117	0.0065	0.0131	0.0077	0.0141	0.0085	0.0107	0.0079	0.0106	0.0060	0.0006	0.0069
**M1T4**	0.0166	0.0692	0.0483	NA	0.0065	0.0116	0.0120	0.0258	0.0100	0.0065	0.0070	0.0069	0.0074	0.0056	0.0000	0.0057
**M1T5**	0.0306	0.0859	0.0117	0.0065	NA	0.2582	0.0149	0.0601	0.0134	0.0080	0.0105	0.0063	0.0128	0.0089	0.0009	0.0035
**M1T6**	0.0232	0.1744	0.0065	0.0116	0.2582	NA	0.0315	0.1479	0.0092	0.0057	0.0062	0.0046	0.0082	0.0138	0.0036	0.0024
**M1T7**	0.0080	0.0093	0.0131	0.0120	0.0149	0.0315	NA	0.0206	0.0072	0.0069	0.0078	0.0072	0.0072	0.0074	0.0000	0.0043
**M1T8**	0.0095	0.0571	0.0077	0.0258	0.0601	0.1479	0.0206	NA	0.0055	0.0081	0.0045	0.0042	0.0061	0.0120	0.0022	0.0025
**M2T1**	0.0260	0.0122	0.0141	0.0100	0.0134	0.0092	0.0072	0.0055	NA	0.0662	0.0198	0.0113	0.0347	0.0231	0.0032	0.0047
**M2T2**	0.0119	0.0106	0.0085	0.0065	0.0080	0.0057	0.0069	0.0081	0.0662	NA	0.0231	0.0455	0.0754	0.1666	0.0033	0.0433
**M2T3**	0.0139	0.0090	0.0107	0.0070	0.0105	0.0062	0.0078	0.0045	0.0198	0.0231	NA	0.0399	0.0135	0.0060	0.0036	0.0095
**M2T4**	0.0088	0.0070	0.0079	0.0069	0.0063	0.0046	0.0072	0.0042	0.0113	0.0455	0.0399	NA	0.0259	0.0133	0.0061	0.0250
**M2T5**	0.0166	0.0094	0.0106	0.0074	0.0128	0.0082	0.0072	0.0061	0.0347	0.0754	0.0135	0.0259	NA	0.2126	0.0025	0.0319
**M2T6**	0.0090	0.0073	0.0060	0.0056	0.0089	0.0138	0.0074	0.0120	0.0231	0.1666	0.0060	0.0133	0.2126	NA	0.0106	0.0931
**M2T7**	0.0009	0.0021	0.0006	0.0000	0.0009	0.0036	0.0000	0.0022	0.0032	0.0033	0.0036	0.0061	0.0025	0.0106	NA	0.0050
**M2T8**	0.0045	0.0048	0.0069	0.0057	0.0035	0.0024	0.0043	0.0025	0.0047	0.0433	0.0095	0.0250	0.0319	0.0931	0.0050	NA

Supplemental Table.4 Summary of the overlapping clones among different populations from Peryer's patch

Mouse-1	Unique Clone Number	T8 (1535)	T7 (1267)	T6 (9243)	T5 (19906)
**T8&T7**	4	1.37%(21)	1.34%(17)	-	-
**T8&T6**	31	10.49%(161)	-	1.97%(182)	-
**T8&T5**	6	0.78%(12)	-	-	0.09%(18)
**T7&T6**	25	-	19.10%(242)	0.27%(25)	-
**T7&T5**	2	-	0.55%(7)	-	0.07%(14)
**T6&T5**	612	-	-	40.72%(3764)	15.19%(3024)
**T8&T7&T6**	0	-	-	-	-
**T8&T7&T5**	0	-	-	-	-
**T8&T6&T5**	46	19.35%(297)	-	9.80%(906)	2.76%(549)
**T7&T6&T5**	3	-	1.26%(16)	0.26%(24)	0.74%(147)
**T8&T7&T6&T5**	2	0.46%(7)	1.18%(15)	0.28%(26)	0.22%(43)
**Non overlay**	-	67.56% (1037)	76.56% (970)	36.07% (4316)	80.94% (16111)

Mouse-2	Unique Clone Number	T8 (1037)	T7 (1033)	T6 (6898)	T5 (35522)
**T8&T7**	1	0.19%(2)	0.48%(5)	-	-
**T8&T6**	13	5.50%(57)	-	1.13%(78)	-
**T8&T5**	14	2.99%(31)	-	-	0.22%(79)
**T7&T6**	6	-	5.81%(60)	0.14%(10)	-
**T7&T5**	4	-	2.03%(21)	-	0.02%(7)
**T6&T5**	433	-	-	45.80%(3159)	10.77%(3825)
**T8&T7&T6**	1	0.19%(2)	0.19%(2)	0.05%(4)	-
**T8&T7&T5**	0	-	-	-	-
**T8&T6&T5**	26	9.06%(94)	-	6. 89%(475)	0.99%(353)
**T7&T6&T5**	1	-	0.19%(2)	0.07%(5)	0.03%(12)
**T8&T7&T6&T5**	0	-	-	-	-
**Non overlay**	-	82.06% (851)	91.29%(943)	45.91%(3167)	87.96%(31246)

Supplemental Table.5 Primers used for PCR amplification of TCR cDNA library preparation

Primer name	Sequence (5'-3')
RT primer	ATATGGATCCGGCGCGCCGTCGACTTTTTTTTTTTTTTTTTTTTTTT
Second strand primer	ATATCTCGAGGGCGCGCCGGATCCNNNNNNNNNNNNTTTTTTTTTTTTTTTTTTTTTTTT
1st PCR forward primer	ATATCTCGAGGGCGCGCCGGATCC
1st PCR reverse primer	ACACTCTTTCCCTACACGACGCTCTTCCGATCTNHNHNCCTTGGGTGGAGTCACATTT
2nd PCR forward primer	CAAGCAGAAGACGGCATACGAGATAAXXXXXXGTGACTGGAGTTCAGACGTGTGCTCTTCCGATCTATATCTCGAGGGCGCGCCGGATCC
2nd PCR reverse primer	AATGATACGGCGACCACCGAGATCTACACTCTTTCCCTACACGACGCTCTTCCGATCT
